# Genome-wide CRISPR/Cas9 library screen identifies PCMT1 as a critical driver of ovarian cancer metastasis

**DOI:** 10.1186/s13046-022-02242-3

**Published:** 2022-01-15

**Authors:** Jingjing Zhang, Yun Li, Hua Liu, Jiahui Zhang, Jie Wang, Jia Xia, Yu Zhang, Xiang Yu, Jinyan Ma, Masha Huang, Jiahui Wang, Liangzhe Wang, Qian Li, Rutao Cui, Wen Yang, Yingjie Xu, Weiwei Feng

**Affiliations:** 1 Department of Obstetrics and Gynecology, Ruijin Hospital, Shanghai Jiao Tong University School of Medicine, Shanghai, 200025 P.R. China; 2 Department of Biochemistry and Molecular Cell Biology, Shanghai Key Laboratory for Tumor Microenvironment and Inflammation, Shanghai Jiao Tong University School of Medicine, Shanghai, 200025 P.R. China; 3 Department of Nephrology, Renji Hospital, School of Medicine, Shanghai Jiao Tong University, Shanghai, 200127 P.R. China; 4 Department of Pathology, Changzheng Hospital, Second Military Medical University, Shanghai, 200003 China; 5 Center for Brain Science, Shanghai Children’s Medical Center, Department of Anatomy and Physiology, Shanghai Jiao Tong University School of Medicine, Shanghai, 200025 China; 6 Shanghai Research Center for Brain Science and Brain-Inspired Intelligence, Shanghai, 201210 China; 7 Department of Dermatology, Boston University School of Medicine, Boston, MA USA; 8 State Key Laboratory of Oncogenes and Related Genes, Shanghai, 200032 P.R. China

**Keywords:** PCMT1, Metastasis, Extracellular matrix, CRISPR/Cas9, Integrin-FAK-Src, Anoikis

## Abstract

**Background:**

The development of lethal cancer metastasis depends on the dynamic interactions between cancer cells and the tumor microenvironment, both of which are embedded in the extracellular matrix (ECM). The acquisition of resistance to detachment-induced apoptosis, also known as anoikis, is a critical step in the metastatic cascade. Thus, a more in-depth and systematic analysis is needed to identify the key drivers of anoikis resistance.

**Methods:**

Genome-wide CRISPR/Cas9 knockout screen was used to identify critical drivers of anoikis resistance using SKOV3 cell line and found protein-L-isoaspartate (D-aspartate) O-methyltransferase (PCMT1) as a candidate. Quantitative real-time PCR (qRT-PCR) and immune-histochemistry (IHC) were used to measure differentially expressed PCMT1 in primary tissues and metastatic cancer tissues. PCMT1 knockdown/knockout and overexpression were performed to investigate the functional role of PCMT1 in vitro and in vivo. The expression and regulation of PCMT1 and integrin-FAK-Src pathway were evaluated using immunoprecipitation followed by mass spectrometry (IP-MS), western blot analysis and live cell imaging.

**Results:**

We found that PCMT1 enhanced cell migration, adhesion, and spheroid formation in vitro. Interestingly, PCMT1 was released from ovarian cancer cells, and interacted with the ECM protein LAMB3, which binds to integrin and activates FAK-Src signaling to promote cancer progression. Strikingly, treatment with an antibody against extracellular PCMT1 effectively reduced ovarian cancer cell invasion and adhesion. Our in vivo results indicated that overexpression of PCMT1 led to increased ascites formation and distant metastasis, whereas knockout of *PCMT1* had the opposite effect. Importantly, PCMT1 was highly expressed in late-stage metastatic tumors compared to early-stage primary tumors.

**Conclusions:**

Through systematically identifying the drivers of anoikis resistance, we uncovered the contribution of PCMT1 to focal adhesion (FA) dynamics as well as cancer metastasis. Our study suggested that PCMT1 has the potential to be a therapeutic target in metastatic ovarian cancer.

**Supplementary Information:**

The online version contains supplementary material available at 10.1186/s13046-022-02242-3.

## Background

Cancer metastasis is a multistep and highly selective process that involves dissociation of tumor cells from the primary site, anchorage-independent growth, apoptosis evasion, cell migration, invasion of surrounding tissues, intravasation into the circulation, extravasation and colonization to distant sites [1]. During this process, tumor cells are in a dynamic state and susceptible to anoikis in response to extracellular matrix (ECM) detachment. Clearly, the dynamic interplay between tumor cells and the microenvironment critically impacts the process of metastatic progression. However, it is unclear how disseminate solid tumor cells acquire the essential ability to survive under “anchorage-independent” (“spheroid”) growth conditions. We decided to address this question using human ovarian carcinoma cells as a model. Ovarian cancer cells shedding from the primary site are able to survive in vivo single cells or free-floating clumps in the ascites fluid, which may be a consequence of anoikis resistance [2, 3].

The ECM is an important component of the tumor microenvironment, and its deposition, degradation, and remodeling are closely associated with cancer development and progression [4]. In addition to tumor stromal cells, tumor cells also secrete numerous ECM components, primarily collagens, fibronectin, laminins, hyaluronan, tenascin C, and periostin, which are highly expressed in metastatic tumors and contribute to building tumor metastasis niches [5–9]. Beyond their well-known functions as structural scaffolds, ECM components are also located between cells and mediate cell–cell interactions [10]. Many of these proteins bind to integrins and their specific cell surface receptors and evoke cell survival or anoikis-counteracting functions by activating downstream signaling, including focal adhesion kinase (FAK), Src kinase, integrin-linked kinase (ILK), PI3K/Akt and mitogen-activated protein kinase (MAPK). The role of the ECM in tumor metastatic progression, especially its deposition, posttranslational modification and reorganization, as well as its dynamic interaction with surface receptors on cancer cells are tightly regulated by biochemical and mechanical cues.

To identify proteins required for anoikis resistance and cancer progression that might be therapeutic targets, we carried out genome-wide a CRISPR/Cas9 knockout screening in ovarian cancer cells under ultralow-attachment conditions that mimic the situation where cancer cells shed from the primary attachment site. Among the genes identified, protein-L-isoaspartate (D-aspartate) O-methyltransferase (*PCMT1*) was found to be the most highly associated with anoikis resistance in ovarian cancer. PCMT1, also known as PIMT, is a repair enzyme that initiates the conversion of spontaneously isomerized aspartyl residues to their normal configuration [11]. Spontaneous protein deamidation and isomerization of normal L-aspartyl and L-asparaginyl residues, generating abnormal L-isoaspartyl (isoAsp) residues, is associated with cellular aging and enhanced by microenvironmental stress [12]. By initiating the conversion of L-isoAsp (or D-Asp) residues to L-Asp residues, PCMT1 has been reported to play a protective role in cells through mechanisms such as repairing proteins involved in apoptosis and protecting certain neuronal cells against Bax-induced apoptosis [13]. Deletion of the *Pcmt1* gene in mice leads to isoAsp accumulation in all tissues measured, especially in the brain. These mice exhibit profound neuropathology and die prematurely from epileptic seizures [14]. Recent evidence documents that *PCMT1* is an unfavorable prognostic predictor and functions as an oncogene in various cancers, including bladder cancer [15] and lung adenocarcinoma [16], indicating a role for PCMT1 in cancer progression. However, the precise role of PCMT1 in cancer development and progression has not been elucidated.

In this study, we identified PCMT1, a key enzyme in protein repair, as a critical driver of cancer anoikis resistance. Importantly, we first linked PCMT1 with the ECM protein LAMB3 and demonstrated that the PCMT1-LAMB3 interaction activates the integrin-FAK-Src pathway to promote cancer cell adhesion, migration and invasion and that these effects can be reversed by treatment with a PCMT1-blocking antibody. Moreover, PCMT1 enhanced ascites formation and distant metastasis in a xenograft mouse model. PCMT1 profiling in human ovarian cancer specimens revealed significantly increased PCMT1 expression in metastases compared with primary tumors.

## Methods

### Patients and tissue samples

Primary ovarian tumor tissues and metastatic tumor tissues were obtained from patients treated at Ruijin Hospital, Shanghai Jiao Tong University School of Medicine between 2018 and 2020 were examined. All patients were diagnosed with ovarian cancer by pathologist and did not receive chemotherapy or radiotherapy before surgery. The tissues which were used to extracted RNA stored in RNAlater solution at -80 °C and the other tissues were made to paraffin blocks for HE and immunohistochemistry (IHC) analysis. The general clinical data of the patients are shown in Table 1. All experiments were conducted with the approval of the Committees for Ethical Review of Research involving Human Subjects at Shanghai Jiao Tong University School of Medicine. The experiments were undertaken with the understanding and written consent of each subject. The study methodologies conformed to the standards set by the Declaration of Helsinki.

**Table 1:** Clinicopathological characteristics of trial patients according to PCMT1 expression

Characteristic	Patients	PCMT1 expression		
	**NO**	**High**	**Low**	***P*-Value**
**All patients**	72	50	22	
Age (years)				0.7175
≤ 50	24	16	8	
> 50	48	34	14	
**Primary tumor size (cm)**				0.7677
≤ 5	18	12	6	
> 5	54	38	16	
**Metastatic tumor size (cm)**				**0.0466**
≤ 5	64	42	22	
> 5	8	8	0	
**Lymphovascular invasion**				0.6873
Positive	12	8	4	
Negative	60	35	25	
**FIGO stage**				**0.0386**
I/II	18	9	9	
III/IV	54	41	13	
**Histology Diagnosis**				0.3455
serous	68	49	19	
Others	4	2	2	

### Cell culture

The human ovarian cancer cell line SKOV3 and Hela was purchased from the Cell Bank of the Chinese Academy of Sciences (Shanghai, China). The human ovarian cancer cell line OVCAR3 was purchased from American Type Culture Collection. SKOV3 cells were cultured in McCoy's 5A (Gibco, USA) supplemented with 10% fetal bovine serum (FBS). HeLa cells were cultured in Dulbecco's Modified Eagle Medium (DMEM) medium (HyClone, USA) supplemented with 10% fetal bovine serum (FBS). OVCAR3 cells were cultured in Roswell Park Memorial Institute (RPMI)-1640 medium (HyClone, USA) supplemented with 0.01 mg/ml bovine insulin and 20% FBS. All of the above media contained 100 U/ml penicillin and 100 μg/ml streptomycin. All cells were cultured in a humidified incubator at 37 °C with 5% CO_2_.

### Genome-wide CRISPR/Cas9 screen

Human GeCKOv2 CRISPR knockout pooled library was a gift from Feng Zhang (Addgene # 1,000,000,048) [17]. For genome-wide CRISPR screening, 1 × 10^8^ SKOV3 cells were infected with the pooled lentiviral GeCKO v2 library at a multiplicity of infection of 0.3. After 5 days of puromycin selection, the cells were divided into two groups. The control group cells were cultured in normal plates, while the experimental group cells were cultured in ultralow-attachment (ULA) plates (3471, Corning, USA) for spheroid formation for 5 days. Then, genomic DNA from approximately 5 × 10^7^ cells were isolated by a Quick-DNA Midiprep Plus Kit (D4075, Zymo research, USA) and subjected to PCR to construct the sequencing library. The forward primer sequence for sgRNA library amplification and next-generation sequencing (NGS) was 5′-AATGATACGGCGACCACCGAGATCTA CACTCTTTCCCTACACGACGCTCTTCCGATCTATCATGCTTAGCTTTATATATC TTGTGGAAAGGACGAAACACC-3′. The reverse primer was 5′-CAAGCAGAAGACGGCATACGAGATGAAGAAGTGTGACTGGAGTTCAGACGTGTGCTCTTCCGATCTCCGACTCGGTGCCACTTTTTCAA-3′ for the control group and 5′-CAAGCAGAAGACGGCATACGAGATAT TCTAGGGTGACTGGAGTTCAGACGTGTGCTCTTCCGATCTCCGACTCGGTGCC ACTTTTTCAA-3′. Each library was sequenced on 50 million reads, and data were analyzed by RNAi Gene Enrichment Ranking (RIGER) analysis. We ran RIGER through GENE-E from Broad Institute website as specified in the paper [17, 18]. The results were ranked by the Z-score of the genes.

### Gene silencing

For the knockdown assay, cells were seeded in 6-well plates and transfected with 200 nM siRNA oligos using Lipofectamine 2000 (Thermo Fisher Scientific, USA). Seventy-two hours post transfection, whole proteins were harvested for western blot analysis to determine the knockdown efficiency. The siRNA sequences used are listed in Table S1.

### Construction of plasmids and generation of the stable line

cDNAs encoding PCMT1 were reversely transcribed from SKOV3 cells mRNA. PCMT1 was subcloned in pHAGE-HA-Puro or pHAGE-EGFP-Puro lentivirus vector [19] for PCMT1 overexpression. Lentivirus packaging was performed in HEK293T cells by transfection of transfer plasmid (pHAGE-PCMT1-HA or pHAGE-PCMT1-EGFP) together with packaging vectors (pMD2.G and psPAX2) using polyethylenimine (PEI) (Polyscience, USA). After 48 h, the medium supernatant containing lentivirus particles was collected for infection. Polybrene (8 μg/ml) was added simultaneously to increase efficiency. After 48 h of infection, the cells were challenged with 1 μg/ml puromycin (ApexBio, USA) for 3–5 days to obtain positive cells.

### Western blot analysis

For immunoblotting, cells were lysed in mammalian cell lysis buffer (MCLB) buffer (50 mM Tris pH 7.5, 150 mM NaCl, 0.5% IGEPAL® CA-630 (Sigma-Aldrich, I3021, USA) for 30 min on ice, and then protein concentrations were determined by Bradford. Equal amounts of protein were separated by 10% SDS PAGE gels and transferred to nitrocellulose filter membranes (Millipore, USA). After blocking with 5% skim milk in TBST (50 mM Tris–HCl at pH 7.4 and 150 mM NaCl, and 0.1% Tween 20) and then indicated with appropriate primary antibodies. Signals were detected with horseradish peroxidase (HRP)-conjugated secondary antibodies and an enhanced chemiluminescence (ECL) detection system (Sage Creation Science).

The following antibodies were used: PCMT1 (10,519–1-AP, Proteintech; PA5-51,872, Thermo), LAMB3 (sc-133178, Santa Cruz Biotechnology Inc.), Cas9 (14697P, Cell Signaling Technology CST), FAK (05–182, UPSTATE), p-FAK (Tyr397) (611,806, BD and 8556, CST), Src (2123, CST), p-Src (Tyr 416) (6943 T, CST), Akt (pan) (4691 T, CST), p-Akt (Ser473) (9271 T, CST), EGFP (A6455, Thermo), GAPDH (60,004, Proteintech), β-actin (66,009, Proteintech), and HA (66,006, Proteintech).

### Immunoprecipitation followed by mass spectrometry (IP-MS)

IP-MS was performed as previously described [19]. HA-tagged PCMT1 and HA-tagged luciferase stable HEK293 cells from six 15-cm tissue culture dishes at ~ 90% confluence was evenly divided into 2 groups. One group was treated with Dimethyl dithiobispropionimidate (DTBP) (Thermo #20,665), while another was not treated. For the group treated with DTBP, cells were harvested in culture media. The cross linker DTBP was added to a final concentration of 50 mM. After rotating for 15 min, glycine was added to quench the reaction at a concentration of 125 mM. Cells were collected by centrifugation, washed with phosphate buffered solution (PBS) 3 times and subjected to protein extraction as described above. Equal amounts of protein were subjected to immunoprecipitation using α-HA magnetic beads (Thermo Fisher Scientific, USA) (50% slurry) overnight at 4 °C with gentle inversion. After washing, the binding protein complex was eluted by 0.1 M glycine (pH 2.0). Half of the eluted samples were preprocessed and analyzed on an LTQ-Orbitrap Fusion Mass Spectrometer (Thermo Fisher Scientific, USA) coupled with an EASY-nLC 1000 Liquid Chromatograph (Thermo Fisher Scientific, USA).

Bioinformatics and statistical analysis of the original mass spectrometric data were performed using PEAKS Studio based on the UniProt database (20,180,524, 20,349). Kyoto Encyclopedia of Genes and Genomes (KEGG) pathway and GO enrichment were analyzed by “clusterprofiler” package in R language about proteins interacted with PCMT1. STRING (Search Tool for the Retrieval of Interacting Genes/Proteins) network analysis of the 39 PCMT1 interacting proteins found in 293 T cells.

### Quantitative real-time PCR

Total RNA was isolated using an RNA extraction kit (B004D, EZBioscience, USA), followed by reverse transcription to cDNA using the HiScript III RT SuperMix for qPCR Kit (R323-01, Vazyme, China). Then, 100 ng of cDNA was used to measure mRNA levels using Hieff qPCR SYBR Green Master Mix (11201ES08, Yeasen Biotech, China). Finally, qPCR results were analyzed using the 2^−ΔΔCT^ method. The primer sequences used are shown in Table S2.

### Cas9 mRNA preparation

The open-reading frame (ORF) of Cas9 was PCR amplified from the px330 plasmid [20] (Addgene, no: 42230) and subcloned into a vector containing the T7 promoter and untranslated region (UTR). Cas9 protein was tagged by HA at the C-terminus. The vectors were linearized by enzyme digestion and amplified by PCR. The amplicons were further purified and used as templates for in vitro transcription (IVT). The modified mRNA was synthesized as described previously [21]. In brief, IVT was conducted using the HyperScribe T7 High Yield RNA Synthesis Kit (ApexBio, USA) with 1–2 µg template and 6 mM 3′-0-Me-m7G(5′)ppp(5′)G (anti-reverse cap analog, ARCA) (ApexBio, USA), 7.5 mM ATP, 1.5 mM GTP, 7.5 mM 5-methyl-CTP, 7.5 mM pseudo-UTP or other modified nucleotides, including 5’-methoxy-UTP, N1-methylpseudouridine (ApexBio, USA). Reactions were incubated at 37 °C for 2–4 h, followed by DNase (Thermo Fisher Scientific, USA) treatment. Then, 3’ poly(A)-tails were added to the IVT RNA products using a poly(A) tailing kit (ApexBio, USA). mRNA was purified by using the RNA Clean and Concentrator Kit (Zymo research, USA).

### Generation of *PCMT1* knockout cells

The sgRNA of PCMT1 was designed through an online website (benchling, https://www.benchling.com.) (Table S3). sgRNA was prepared by IVT of the T7 promoter and sgRNA targeting sequence containing PCR products. The forward primer for *PCMT1*-sgRNA was 5′- TTAATACGACTCACTATAGGGCGATGGCCTGGAAATCCGGGTTTTAGAGCTAGAAATAGC-3′. The long forward primers were used together with sgRNA_rev (5′-AAAAAGCACCGACTCGGTGCC-3′) to amplify the chimeric sgRNA template from px330 (Addgene no: 42230). The PCR product (sgPCMT1) was used for IVT using the T7 High Yield In Vitro Transcription Kit (E2040S, NEB, USA), and sgRNA was purified using the GeneJet RNA purification kit (K0731, Thermo Fisher Scientific, USA). The sgRNA and cas9 mRNA were co-transfected into SKOV3 cells using Lipofectamine 2000. After 48 h, cells were diluted and seeded in 96-well plates for single clone isolation. After these cell clones grew, genomic DNA and total protein were harvested, and PCR, Sanger sequencing and western blotting were used to detect *PCMT1* knockout.

### Ovarian cancer xenograft tumor model

To prepare an ovarian cancer metastasis model, female BALB/c nude mice (4–5 weeks old) (Shanghai Lingchang Biology Co., LTD, China) were maintained under specific pathogen-free conditions at the School of Medicine, Shanghai Jiao Tong University. The animal licence number for this experiment is SYXK 2018–0027. The mice were randomly divided into 4 groups (*n* = 6 per group) that received inoculation of the following cells: SKOV3-luc cells, SKOV3-PCMT1-HA cells, SKOV3-sgControl cells or SKOV3-sgPCMT1 cells. A total of 3 × 10^6^ cells were inoculated into mice by i.p. injection. Mice were sacrificed at approximately 6 weeks or 10 weeks, and the tumors were harvested, weighed, and photographed. Tumor tissue and affected organ samples were fixed in 4% paraformaldehyde and embedded in paraffin followed by sectioning and staining with hematoxylin and eosin (H&E). The slides were assessed using a Digital Pathology Slide Scanner (KF-PRO-120, KFBIO, China).

### Immunohistochemistry

For immunohistochemistry (IHC) analysis, paraffin-embedded tissues were cut into 4-µm sections. The sections were deparaffinized in xylene and rehydrated through a graded ethanol series and then subjected to antigen repair by citric acid with the microwave boiling method. Endogenous peroxidase activity was blocked by incubation with 3% hydrogen peroxide at room temperature for 10 min. Then, the slides were incubated with anti-PCMT1 antibody (1:50) overnight at 4 °C. The next day, sections were washed in PBS three times for 5 min each. Secondary antibody was applied for 30 min at 37 °C, and color was developed with a diaminobenzidine peroxidase substrate kit (Impact DAB, Vector Laboratories, USA). Sections were then counterstained with hematoxylin, dehydrated and mounted. The protein level was calculated by Image-Pro Plus software.

### Cell proliferation assay

Cell proliferation was detected using the Cell Counting Kit-8 (CCK-8) assay. In brief, cells were seeded at the appropriate density in a 96-well plate and treated as described. Then, the CCK-8 assay was performed using a CCK-8 kit following the manufacturer’s protocol (Apexbio, USA), and then cell proliferation was quantified by detecting the optical density at 450 nm (OD450) on a microplate reader (Tecan, Infinite 200 PRO).

### Wound healing assay

To perform wound healing assays, cells were seeded in a 6-well plate and manually wounded by scraping with pipette tips when they reach confluence. The scratch wound was monitored and photographed at the indicated time points. The distance of cell migration was calculated by ImageJ software.

### Cell adhesion assay

A 96-well plate was precoated with 20 μg/ml laminin (23,017,015, Thermo Fisher Scientific, USA) or 10ug/ml fibronectin (F0895, Sigma, USA) at 4 °C overnight. Then, the plate was washed with medium containing 0.1% BSA 2 times and blocked with medium containing 0.5% BSA at 37 °C in a CO_2_ incubator for 45–60 min. To perform the cell adhesion assay, 2–3 × 10^4^ cells were seeded in each well, and the cells were incubated in a CO_2_ incubator at 37 °C for 30 min. Then, the plate was subjected to shaking at 2000 rpm for 10–15 s, followed by fixation with 4% paraformaldehyde at room temperature for 10–15 min. Subsequently, the adherent cells were stained with crystal violet for 10 min and then photographed. Finally, cells were treated with 2% SDS and incubated at room temperature for 30 min, and then the absorbance was detected at a wavelength of 590 nm using a microplate reader (Tecan, Infinite 200 PRO).

### Cell immunofluorescence

To detect the subcellular localization of proteins, OVCAR3 and SKOV3 cells were plated onto coverslips in 6-well plates and grown overnight to 50–60% confluence. Cells were washed with PBS and fixed with 4% paraformaldehyde for 15 min at room temperature and permeabilized with 0.2% Triton X-100 for 10 min. After that, cells were washed with PBS 3 times and blocked with PBS blocking buffer containing 2% normal goat serum and 2% BSA for 1.5 h at room temperature. Then, the cells were incubated with the corresponding antibody (1:200 dilution in blocking buffer) at room temperature for 1 h, washed with PBS and incubated with Alexa Fluor 488 secondary antibody (Molecular Probe, USA) at a 1:400 dilution for 1 h. Finally, the cells were washed with PBS, stained with 4,6-diamidino-2-phenylindole (DAPI) and mounted on slides with Prolong Gold antifade mounting medium (Thermo Fisher Scientific, USA). The fluorescence images were captured by a fluorescence microscope (Leica DMi8, Germany).

### Live-cell imaging

Fluorescence imaging of live cells was performed using an oil objective on a microscope (Leica DMi8, Germany) housed in a closed system to maintain the temperature at 37 °C and CO_2_ levels at 5%. Cells were seeded in glass-bottom dishes, and the media were replaced with phenol red-free media (Thermo Fisher Scientific, USA) before photography. Fluorescent images were then captured every 5 min for 4 h using Leica Application Suite X Imaging software.

### Spheroid formation assay

For the spheroid formation assay, cells were cultured in a 6-well ULA plate (3471, Corning, USA). After 3 days, spheroid formation was monitored and photographed. After culturing for 5 days under ULA conditions, the number of living cells was determined using trypan blue staining.

### Cell Transwell assay

To conduct the Transwell invasion assay, 3–5 × 10^4^ cells precultured in serum-free medium were seeded into the top of Transwell chambers with 8-µm pores (Costar, Cambridge, MA, USA) precoated with Matrigel matrix (1:8 dilution in serum-free medium; BD 356,234, USA). Then, the chambers were placed into 24-well plates containing medium with 10% serum. Forty-eight hours later, the cells were fixed and stained with 0.1% crystal violet. Five fields of vision were randomly photographed for cell counting.

### Determine the secretion of endogenous PCMT1

To determine the endogenous secretion of PCMT1, sgControl and sgPCMT1 SKOV3 cells were seeded in 10 cm dishes respectively. The cells were normally cultured to a density of 80% and incubated with FBS-free medium for 48 h. Then 8 ml of the culture supernatant was collected and filtered by a 0.22um filter (Merk Milipore, USA). The resulting samples were subjected for ultrafiltration over a 10 kDa cut-off membrane using centrifugal columns (Merk Milipore, USA) to extract the majority of secreted proteins. Finally, proteins were quantified and subject to western blot analysis.

### Stastistics

All statistical analyses were performed using GraphPad. Graphed data represent the means ± standard deviation from at least three independent experiments. Two-tailed, paired Student’s *t*-test was used to determine the statistical significance unless otherwise specified.

## Results

### A genome-wide pooled sgRNA library screen in an ovarian cancer cell metastasis model

**Fig. 1:**
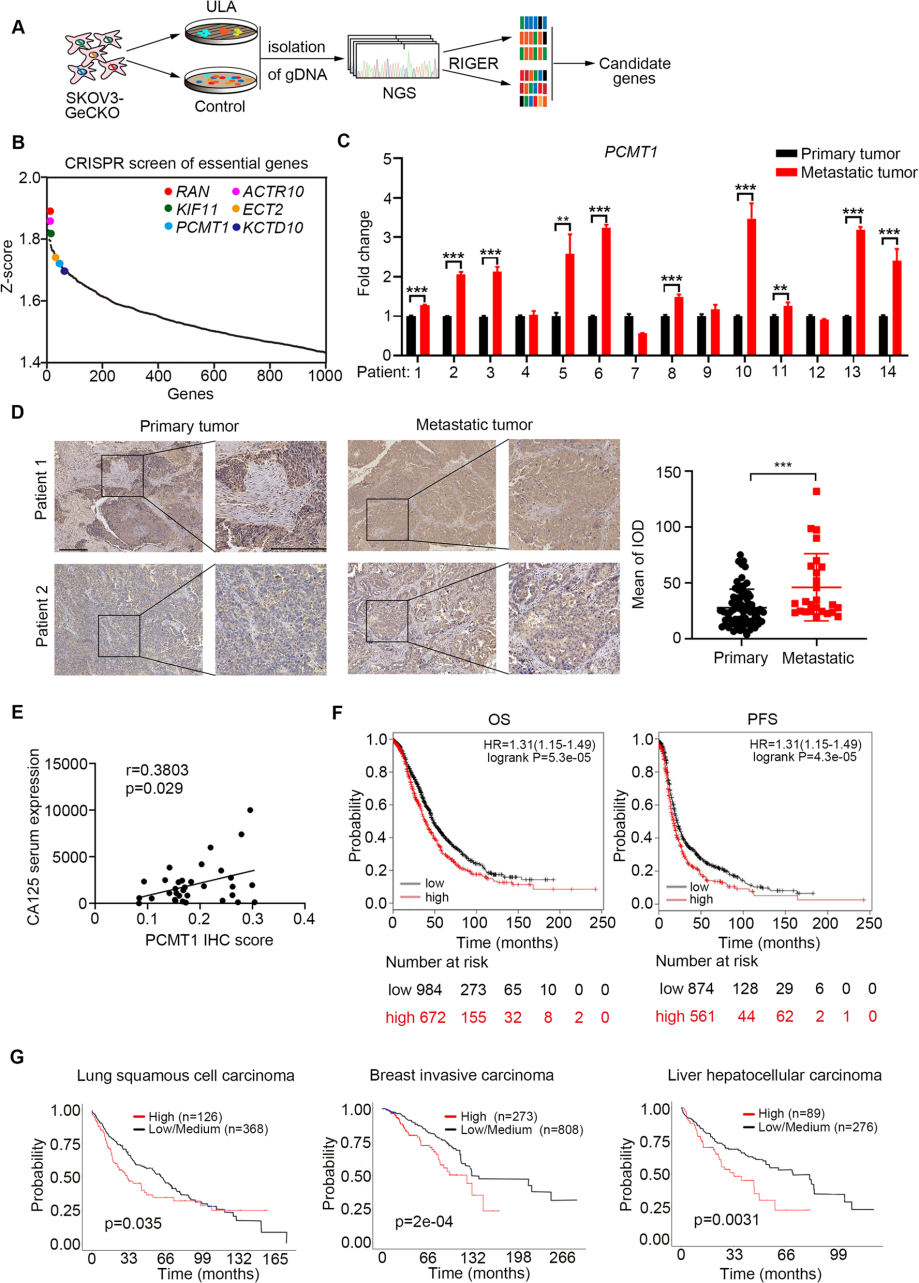
A pooled genome-wide CRISPR screen in an ovarian cancer cell metastasis model. **A** Schematic diagram of the genome-wide CRISPR screen in normal monolayer culture and in ultralow-attachment (ULA) plates condition. **B** A genome-scale CRISPR/Cas9 genetic screen identified the potential genes required for anoikis resistance. Genes were rank-ordered by Z score calculated using RIGER analysis. Candidate genes and previously reported genes related to anoikis resistance are highlighted. **C** Validation of *PCMT1* mRNA expression levels in 14 pairs of in situ ovarian cancer tissues and metastatic cancer tissues. **D** Representative IHC images (left) and semiquantitative evaluation of immunohistochemical expression of PCMT1 (right) in primary tumors (*n* = 72) and metastatic tumors (*n* = 26). **E** The correlation between PCMT1 expression and CA125 serum expression. **F** Overall survival (OS) (*n* = 1656, low expression: 984; high expression: 672) and progression-free survival (PFS) (*n* = 1435, low expression: 874; high expression: 561) were summarized in the high and low *PCMT1* expression groups by using Kaplan–Meier survival curves in ovarian cancer. **G** The survival curves of the high and low *PCMT1* expression groups in different cancer types were analyzed by ULCAN website. (scale bar: 250 μm; Data were statistically analyzed with Student's t-test and value was shown as mean ± SD of 3 independent experiments; *P < 0.05; **P < 0.01; ***P < 0.001.)

To systematically identify the critical genes associated with anoikis resistance in ovarian cancer, we performed a pooled genome-wide CRISPR/Cas9 knockout screen in ovarian cancer. The human GeCKO v2 CRISPR library contains 123,411 unique sgRNAs targeting 19,050 protein-coding genes and was used to generate a mutant ovarian cancer cell pool by transduction of SKOV3 cells at a low multiplicity of infection (MOI = 0.3) followed by puromycin selection [17] (Fig. S1A and B). We then cultured the SKOV3-GeCKO cell pool in normal monolayer culture or in ultralow-attachment (ULA) plates for 5 days to enable negative and positive screening. We hypothesized that knockout of anoikis resistance driver genes will sensitize ovarian cancer cells to detachment-induced cell death and that these cells will fail to form spheroids. In addition, we hypothesized that under ULA culture conditions, cells carrying sgRNA targeting anoikis resistance genes will be negatively selected, and their corresponding sgRNA will be depleted in the library. The genomic DNA was harvested from 5 × 10^7^ cells, and the integrated sgRNA cassettes were amplified from genomic DNA by PCR and subjected to massively parallel Illumina sequencing (Fig. 1A). According to the fold change and the number of each sgRNA, the genes which fold change is greater than 2 and the number of sgRNA greater than 3 were showed (Fig. S1C). We identified 286 genes from negative screen and 122 genes from positive screen (Fig. S1C). RNAi Gene Enrichment Ranking (RIGER) statistical algorithms were used to rank screening hits by the consistent enrichment among multiple sgRNAs that target the same gene [17]. This analysis gave a ranking list based on the Z score to systematically identify genes that were positively or negatively selected in the ULA-cultured population. From this CRISPR/Cas9 knockout library screening, we identified a subset of sgRNAs targeting 1238 genes that were significantly depleted (Z score > 1.4) in the ULA cultured cells when compared to the monolayer cultured control cells, indicating that these genes might be associated with anoikis resistance and spheroid formation (Fig. 1B). Gene Ontology (GO) pathway analysis suggested that the most enriched functional categories for the essential gene set fall into several categories: protein repair, translational initiation, microtubule-based movement, and intracellular protein transport (Fig. S1D). In addition, several previously reported genes related to anoikis resistance were identified, including *RAN*, *KIF11*, and *ECT2* (Fig. 1B) [22–24], providing internal validation of this approach.

Of the top candidate genes, *KCTD10*, *ACTR10*, *RIF1*, *TECB1*, *PCMT1*, *RBM8A*, *PSMD14* and *FAM32A* were selected for clinical validation by comparing the mRNA levels in paired patient specimens, including primary serous ovarian cancer and metastatic tumor tissues. By carrying out quantitative real-time PCR (qRT-PCR), we found that, compared with those in the corresponding primary serous ovarian cancer, the mRNA levels of *KCTD10*, *PCMT1*, and *ACTR10* were significantly increased in metastatic tumor tissues (Fig. S1E). We next increased the sample size to verify the expression of genes in ovarian cancer. The results showed that compared with that in the corresponding primary serous ovarian cancer, the mRNA level of *PCMT1* was increased in metastatic tumor tissue for 10 pairs of patients (Fig. 1C and Fig. S1F). To determine the significance of PCMT1 in epithelial ovarian cancer (EOC) progression, we compared the expression of PCMT1 in 72 primary tumor tissues and 26 metastatic tumor tissues by IHC analysis. A representative picture of paired primary and metastatic tumors is shown in Fig. 1D. Cytoplasmic and nuclear PCMT1 expression was significantly higher in metastatic tumors than in primary tumors (Fig. 1D). The relationships between clinicopathological factors and PCMT1 expression in patients with ovarian cancer are shown in Table 1. Our data indicated that PCMT1 expression was positively related to the clinical stage of ovarian cancer (*P* = 0.0386) and metastatic tumor size (*P* = 0.0466), whereas it was not correlated with patient age (*P* = 0.7175), primary tumor size (*P* = 0.7677), or tumor histology type (*P* = 0.3455) (Table 1). We also found that the expression of PCMT1 was associated with the serum expression of CA125, which is the biomarker of progression in ovarian cancer (Fig. 1E). Furthermore, we performed Kaplan–Meier survival analyses of *PCMT1* mRNA expression data from The Cancer Genome Atlas (TCGA) dataset. High *PCMT1* mRNA expression was associated with poor overall survival (OS) (*n* = 1656, low expression: 984; high expression: 672, logrank *P* = 5.3e-05) and progression-free survival (PFS) (*n* = 1435, low expression: 874; high expression: 561, logrank *P* = 4.3e-05) in patients with EOC (Fig. 1F). Moreover, we found that high expression of *PCMT1* indicated poor prognosis in invasive breast (*p* = 2e-04), lung squamous cell (*p* = 0.035) and liver (*p* = 0.0031) cancers as shown in Kaplan–Meier survival curves from TCGA dataset (Fig. 1G), which further indicated that PCMT1 may play important roles in cancer progression.

### PCMT1 is critical for cell adhesion, invasion and migration in vitro

**Fig. 2:**
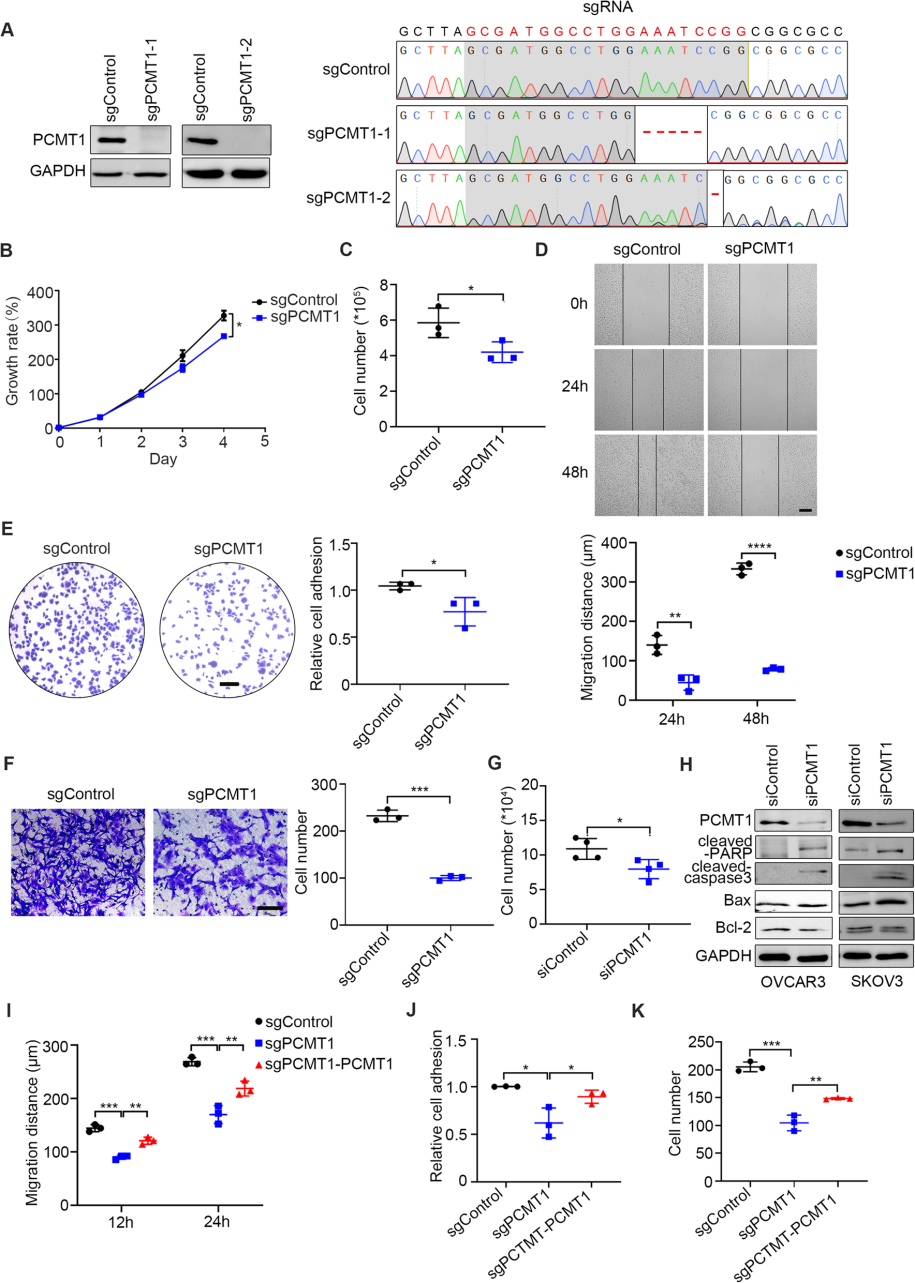
PCMT1 contributes to metastasis-associated tumor cell phenotypes. **A** PCMT1 knockout in SKOV3 cells was verified via western blotting (left) and DNA sequencing (right). **B** Cell proliferation of the control and PCMT1-knockout SKOV3 cells was assessed every 24 h for 5 days using the CCK-8 assay. **C** The living cell counts of control SKOV3 and PCMT1 knockout SKOV3 cells after a 5-day spheroid formation assay. **D** Wound healing assays of control SKOV3 and PCMT1 knockout SKOV3 cells were performed at 0, 24, and 48 h after the cells were scratched. Up, representative images, down, quantification of migration distance. **E** Representative images of the cell adhesion assay (left) and analyses of adhesion capacity (right) comparing control SKOV3 and PCMT1 knockout SKOV3 cells (using laminin). **F** Representative images and quantification of the cell invasion assay comparing control SKOV3 and PCMT1 knockout SKOV3 cells. **G** Over 5 days of spheroid formation in control OVCAR3 cells and PCMT1-silenced OVCAR3 cells, the numbers of viable cells were counted after trypsin digestion and trypan blue staining. **H** After ULA cultured for 72 h, the amounts of apoptotic-antiapoptotic proteins were examined in PCMT1 silenced OVCAR3 and SKOV3 cells. Quantification of cell migration distance (**I**), cell adhesion capacity (using laminin) (**J**), and cell invasion (**K**) in the control, PCMT1-knockout and PCMT1-reexpressing SKOV3 cells. (scale bar: 200 μm; Data are shown as mean ± SEM of 3 independent experiments. Data were statistically analyzed with Student's t-test. *P < 0.05; **P < 0.01; ***P < 0.001.)

The impact of PCMT1 loss on EOC cell invasiveness was examined in both *PCMT1* knockout and knockdown conditions. We used CRISPR/Cas9 technology to generate the *PCMT1* knockout (KO) SKOV3 cell line, and both western blotting and genomic DNA sequencing were performed to verify *PCMT1* knockout (Fig. 2A). We observed that *PCMT1* knockout cells showed reduced cell proliferation, as measured by the CCK-8 assay (Fig. 2B). Compared with the control cells, PCMT1-deficient cells displayed markedly decreased viability during spheroid formation under ULA condition (Fig. 2C).

Cancer cells develop anoikis resistance to facilitate their invasion to other sites for distant metastasis. Since we have demonstrated that PCMT1 is involved in anoikis resistance, we asked whether it could also affect cell migration, adhesion and invasion in ovarian cancer cells. Collective cell migration capacity was evaluated by wound healing assays. To eliminate the effect of cell proliferation, the cells were cultured under serum starvation conditions after a scratch was made. We found that knocking out *PCMT1* weakened cell migration, as indicated by reduced wound closure distance (Fig. 2D). To evaluate the effect of PCMT1 on the adhesion behavior of ovarian cancer cells, adhesion assays using plates coated with laminin/fibronectin (FN) were performed. The results showed that cell adhesion was significantly decreased when PCMT1 expression was lost (Fig. 2E and Fig. S2A). Consistently, the transwell assay revealed that knocking out PCMT1 inhibited cell invasion in vitro (Fig. 2F). Next, we knocked down *PCMT1* with siRNA in OVCAR3 cells and performed a cell spheroid formation assay. siRNA silencing of *PCMT1* effectively reduced PCMT1 expression, as verified by western blotting and qRT-PCR (Fig. S2B). We observed that the cell spheroid formation ability was decreased, and the spheroids were smaller and more loosely distributed when *PCMT1* was knocked down (Fig. S2C). In addition, viable cell counting showed that the number of live cells decreased after *PCMT1* knockdown in the 5-day spheroid formation assay (Fig. 2G). Next, we analyzed the behavior of proapoptotic–antiapoptotic proteins under ultralow culture condition. In SKOV3 and OVCAR3 cells, silencing of PCMT1 induced apoptosis, as indicated by increased cleavage of caspase-3 and its substrate PARP-1. However, under the same condition, the levels of Bcl-2 and Bax did not change significantly, suggesting that intrinsic apoptosis pathway was not activated when cells were cultured at ultra-low condition for 72 h (Fig. 2H). Similar results were found in PCMT1 knockout SKOV3 cells (Fig. S2D). In line with these findings, knocking down *PCMT1* inhibited cell migration (Fig. S2E-G) and cell adhesion (Fig. S3A and B) in cervical cancer Hela cells. These results indicated the pro-invasive effect of PCMT1 in multiple gynecological cancer cell lines.

To further investigate the function of PCMT1 in ovarian cancer, we transfected the *PCMT1*-KO cells with either empty vector (control) or PCMT1-HA for a rescue experiment (Fig. S3C). PCMT1 re-expression in *PCMT1*-KO cells was able to significantly increase the speed of cell migration during wound closure (Fig. 2I and Fig. S3D), cell adhesion (Fig. 2J and Fig. S3E), cell invasion (Fig. 2K) and cell proliferation (Fig. S3F) of *PCMT1*-KO cells. Taken together, our experiments demonstrated that *PCMT1* knockout inhibited metastasis-relevant traits of ovarian cancer cells in vitro and that re-expression of PCMT1 in *PCMT1*-KO cells rescued the phenotype.

**Fig. 3:**
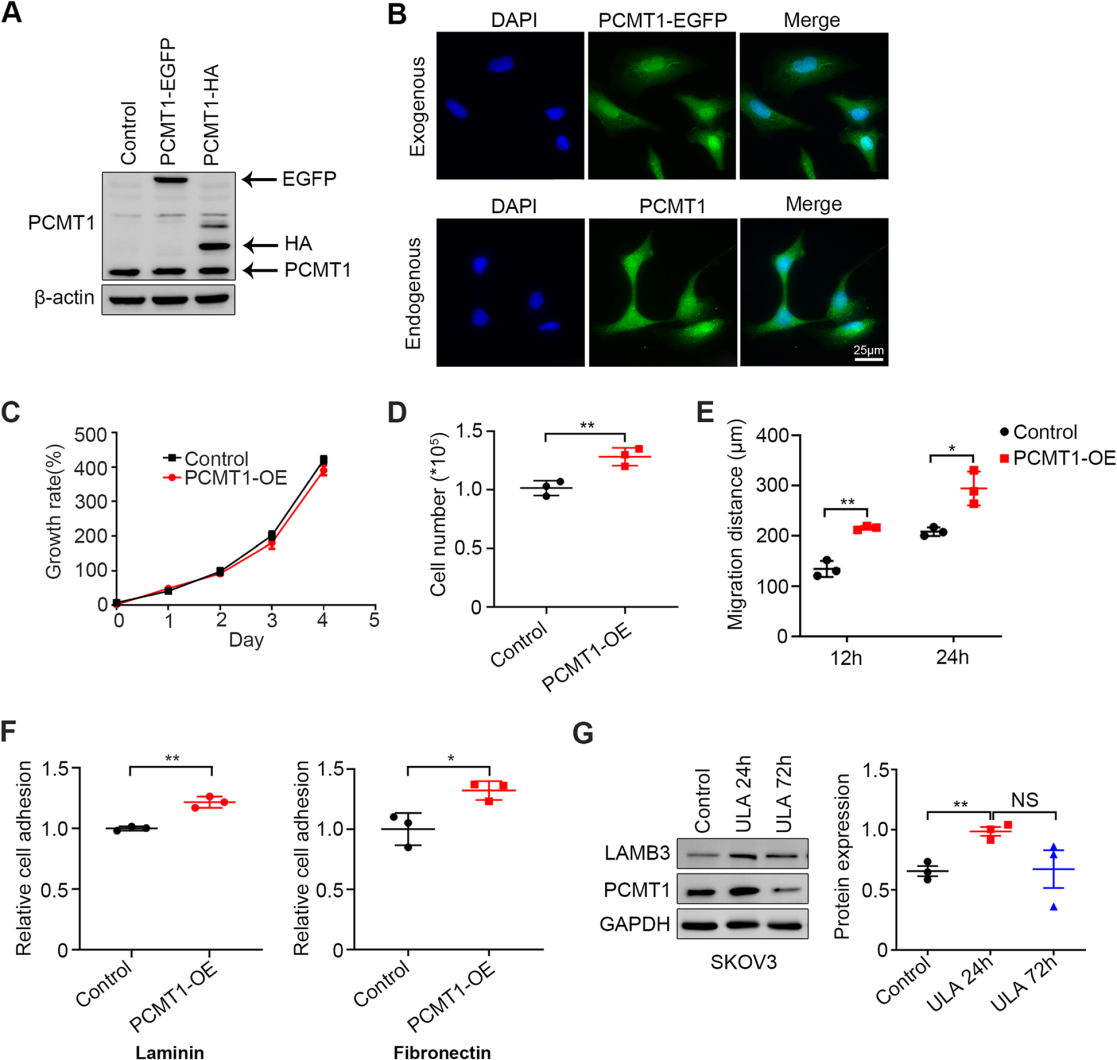
PCMT1 is critical for cell adhesion, invasion and migration in vitro. **A** The protein expression levels of EGFP- or HA-tagged PCMT1 were verified by western blotting as indicated. **B** Representative immunofluorescence showing the localizations of ectopic expression of PCMT1 (upper) and endogenous PCMT1 (lower) in SKOV3 cells. **C** SKOV3 cell proliferation of the control group and PCMT1-OE group was tested for 5 days by CCK-8 assay. **D** Quantification of the number of living cells after OVCAR3 cell spheroid formation for 5 days between the control group and the PCMT1-OE group by cell count assay. **E** Quantification of the cell migration distance of both SKOV3 control and PCMT1-OE cells observed at 12 h and 24 h after wounding. **F** Quantification of SKOV3 cell adhesion in the control or PCMT1-OE group under laminin-coated (left) or fibronectin-coated (right) conditions after 30 min of attachment (scale bar: 25 μm *P < 0.05; **P < 0.01; ***P < 0.001 *n* = 3.) (**G**) SKOV3 cells were plated in ULA plates, and the amounts of LAMB3 and PCMT1 were determined by western blotting at 24 h and 72 h after seeding under ULA conditions (scale bar: 200 μm; Data are shown as mean ± SEM of 3 independent experiments. Data were statistically analyzed with Student's t-test. *P < 0.05; **P < 0.01; ***P < 0.001)

The effect of PCMT1 on the adhesion and migration ability of EOCs was further examined by generating stable PCMT1-HA transfectants in SKOV3 and OVCAR3 cells (Fig. 3A and Fig. S4A). We found that PCMT1 was located in the cell cytoplasm and nucleus by observing EGFP-tagged PCMT1 or immunofluorescent staining endogenous PCMT1 (Fig. 3B and Fig. S4B). When cells were cultured in monolayer conditions, the CCK-8 assay suggested that overexpression of PCMT1 had no effect on SKOV3 and OVCAR3 cell viability (Fig. 3C and Fig. S4C). However, when these cells were placed in a detached environment, PCMT1-overexpressing (PCMT1-OE) OVCAR3 cells showed enhanced spheroid formation and increased cell viability compared with the control group (Fig. 3D), suggesting that the presence of PCMT1 counteracts anoikis. The phenotype was not observed in the SKOV3 cell line, which may be because the basal level of PCMT1 in SKOV3 cells was high (Fig. S4D). Furthermore, we found that upregulation of PCMT1 expression markedly promoted cell migration, as detected by wound healing assay (Fig. 3E). In addition, an adhesion assay was used to detect the role of PCMT1 on moment attachment. The results indicated that a high level of PCMT1 enhanced cell adhesion, suggesting that escaped cells from the initial tumor with high PCMT1 expression could quickly attach to the other site and consequently develop implantation metastasis (Fig. 3F and Fig. S4E). Taken together, our experiments demonstrated that PCMT1 expression is essential for maintaining the metastatic traits of ovarian cancer cells in vitro.

The above results indicated that PCMT1 plays an important role in anoikis resistance; thus, we determined whether the expression of PCMT1 would change during cell detachment. We discovered that the expression of PCMT1 was increased under ULA culture conditions for 24 h and then decreased to basal levels after ULA culture for 3 days (Fig. 3G). This result meant that PCMT1 played an important role in the process of tumor cell shedding from the primary site. Taken together, our CRISPR screen, clinical specimen analysis, and *PCMT1* knockdown/knockout validation identified that PCMT1 was essential to anoikis resistance in ovarian cancer.

### Immunoprecipitation followed by mass spectrometry (IP-MS) identified that PCMT1 interacts with LAMB3

**Fig. 4:**
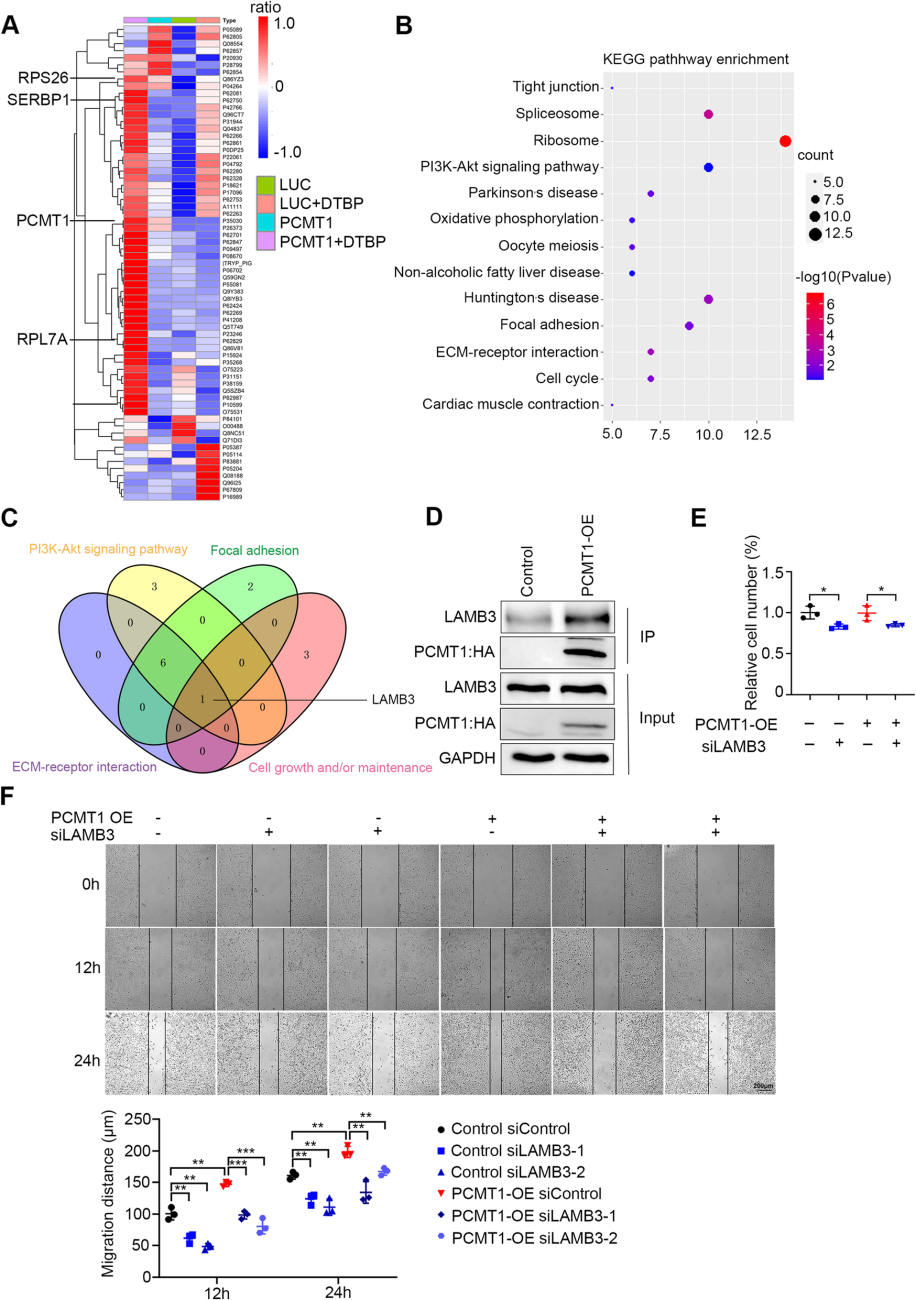
IP-MS indicates that PCMT1 interacts with LAMB3. **A** The heatmap of identified interacting proteins in our four IP-MS samples groups. **B** KEGG pathway enrichment of interacting with the PCMT1 protein set. **C** The proteins identified by IP-MS were subjected to KEGG-based clustering analysis. **D** IP-western blot analysis validated the interaction of PCMT1 and LAMB3. **E** The number of living cells after spheroid formation was calculated by cell count assay in control or *LAMB3*-silenced PCMT1-OE SKOV3 cells. **F** *LAMB3* was knocked down in control or PCMT1-OE SKOV3 cells, and cell migration was determined and quantified. (scale bar: 200 μm; Data are shown as mean ± SEM of 3 independent experiments. Data were statistically analyzed with Student's t-test. *P < 0.05; **P < 0.01; ***P < 0.001)

To gain insight into the mechanism by which PCMT1 promotes cell metastasis traits in ovarian cancer, we performed IP-MS. The crosslinking agent DTBP was used to capture transient protein–protein interactions. Tandem immunoprecipitation using HEK293 cells overexpressing C-terminal HA-tagged PCMT1 or luciferase (luc) as bait was performed with 4 different groups: SKOV3-luc-HA; SKOV3-luc-HA + DTBP; SKOV3-PCMT1-OE; and SKOV3-PCMT1-OE + DTBP. The results showed that 216 proteins interacted with PCMT1 in the SKOV3-PCMT1-OE group when nonspecific binding proteins were excluded in the control group, while in cells that were treated with DTBP, 302 proteins were identified to interact with PCMT1 (Fig. S5A). Taking the intersection of the two groups, 39 proteins were identified as high-confidence PCMT1-interacting proteins (Fig. S5A); therefore, we subjected these proteins to an analysis of known protein–protein interactions with the Search Tool for the Retrieval of Interacting Genes/Proteins (STRING) database. The results showed that the PCMT1-interacting proteins we identified have not been reported (Fig. S5B). Among all the identified interacting proteins, proteins associated with cell–cell adhesion were highly enriched in PCMT1 overexpressed group, such as RPS26, RPL7A, PCMT1, SERBP1 protein (Fig. 4A). To identify the specific PCMT1-interacting protein associated with cell metastasis traits, we performed biogenic analysis on proteins interacting with PCMT1. Kyoto Encyclopedia of Genes and Genomes (KEGG) pathway enrichment analysis of high-confidence PCMT1-interacting proteins indicated that they are involved in ECM-receptor interaction, focal adhesion (FA) and the PI3K-Akt signaling pathway (Fig. 4B). The GO analysis showed that proteins interacting with PCMT1 were enriched in cell–cell adhesion interactions (Fig. S5C). According to the bioinformatics analyses, we hypothesized that the protein interacting with PCMT1 in these pathways may affect cell adhesion and migration. Therefore, the common proteins in the ECM-receptor interaction, FA, cell growth and/or maintenance and PI3K-Akt signaling pathways were assessed. As a result of this analysis, we identified the laminin subunit Beta 3(LAMB3) protein was identified as a potential downstream protein of PCMT1 (Fig. 4C).

Laminins are heterotrimeric proteins of the ECM and a major component of the basal lamina. They critically contribute to multiple cellular functions, including cell differentiation, migration, adhesion, proliferation and survival [25, 26]. Upon cell detachment, the mechanism of anoikis resistance can be summarized via three major pathways: inhibition of the death receptor-mediated pathway, activation of the ECM-integrin cell survival pathway and inhibition of the mitochondrial-mediated death pathway [27]. Based on our IP-MS results, we assumed that PCMT1 may interact with LAMB3 and affect cell migration and adhesion. Therefore, we first performed immunoprecipitation experiments followed by western blotting in SKOV3 ovarian cancer cells. Consistent with the IP-MS results, PCMT1 pulled down LAMB3 in SKOV3 cells, indicating that they interact (Fig. 4D). This finding was further supported by immunofluorescence staining which indicated co-localization of these two proteins (Fig. S5D). To investigate whether PCMT1 plays roles in ovarian cancer through LAMB3, we knocked down *LAMB3* in control and PCMT1-OE cells with specific siRNA targeting *LAMB3*. As shown in Fig. 4E and F, silencing of *LAMB3* reduced the cell spheroid formation and migration of SKOV3 cells with PCMT1 overexpression, suggesting that LAMB3 acts downstream of PCMT1.

### Cell invasion and adhesion are enhanced by supernatant from PCMT1-OE cells and inhibited by a PCMT1 blocking antibody

**Fig. 5:**
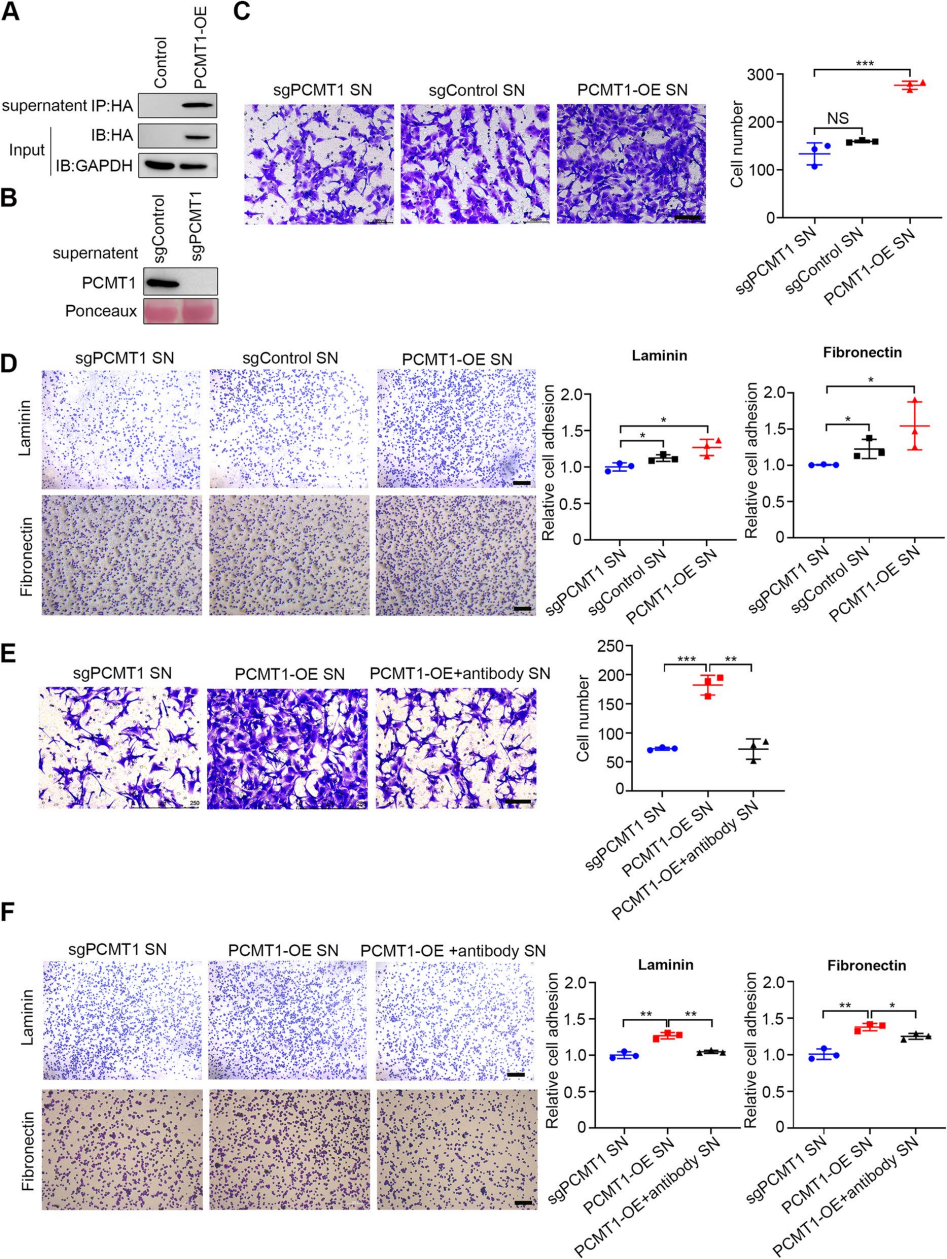
Cell invasion and adhesion are enhanced by supernatant from PCMT1-OE cells and inhibited by a PCMT1 blocking antibody. **A** The culture supernatant of PCMT1-OE SKOV3 cells was collected and immunoprecipitated using an anti-HA antibody, followed by western blot analysis. **B** The culture supernatant of sgControl and sgPCMT1 SKOV3 cells were collected and subjected for analysis of PCMT1 expression by western blot. **C-D** The media of *PCMT1* knockout cells were replaced with the supernatants (SN) from *PCMT1*-KO (sgPCMT1) SKOV3 cells, control (sgControl) SKOV3 cells and PCMT1-OE cells. The cells were then continuously cultured for 24 h and examined for cell invasion (**C**) and cell adhesion (**D**). **E** and **F** The supernatant of PCMT1-OE SKOV3 cells was collected and incubated with PCMT1 antibody for 4 h and then used for *PCMT1*-KO SKOV3 cell culture. After 24 h, cell invasion (**E**) and adhesion (**F**) were determined (scale bar: 200 μm; Data are shown as mean ± SEM of 3 independent experiments. Data were statistically analyzed with Student's t-test. *P < 0.05; **P < 0.01; ***P < 0.001)

In previous studies, PCMT1 was mainly described as an intracellular and soluble protein [28, 29]. We wanted to explore the role of PCMT1 secreted into the ECM in ovarian cancer. Therefore, we first detected whether PCMT1 could be secreted into the ECM. We collected supernatant from PCMT1-OE cells and performed immunoprecipitation using magnetic HA beads. Western blotting results indicated that there was much PCMT1 secreted in the supernatant from PCMT1-OE SKOV3 cells (Fig. 5A). Meanwhile, the secretion of endogenous PCMT1 was also detected in the culture supernatants of SKOV3 cells (Fig. 5B). To determine whether PCMT1 promotes the invasive potential of EOC through its secreted form, we treated *PCMT1*-KO SKOV3 cells with supernatant collected from PCMT1-OE SKOV3 cells. Transwell assays showed that the number of invading *PCMT1*-KO SKOV3 cells increased when cells were treated with supernatant collected from PCMT1-OE SKOV3 cells (Fig. 5C, P = 0.0005). Similarly, compared to that in the control group, the *PCMT1*-KO cell adhesion ability was enhanced after treatment with supernatant containing PCMT1 (Fig. 5D). However, we did not observe that supernatant from PCMT1-OE cells enhanced *PCMT1*-KO cell migration (Fig. S6A).

To further verify the function of secreted PCMT1, we examined the effect of a blocking antibody against PCMT1. Briefly, PCMT1 blocking antibody was added to PCMT1-OE supernatant at a concentration of 1 µg/ml for 4 h to block secreted PCMT1. Then, the different supernatants were filtered through a 0.22-μm filter and added to *PCMT1*-KO cells for 24 h. In the presence of PCMT1 blocking antibody, there was a significant reduction in cell invasion and adhesion capacity (Fig. 5E and F). These findings suggested that secreted PCMT1 contributes to cancer cell invasion potential.

### PCMT1 regulates the integrin-FAK-Src pathway through LAMB3

**Fig. 6:**
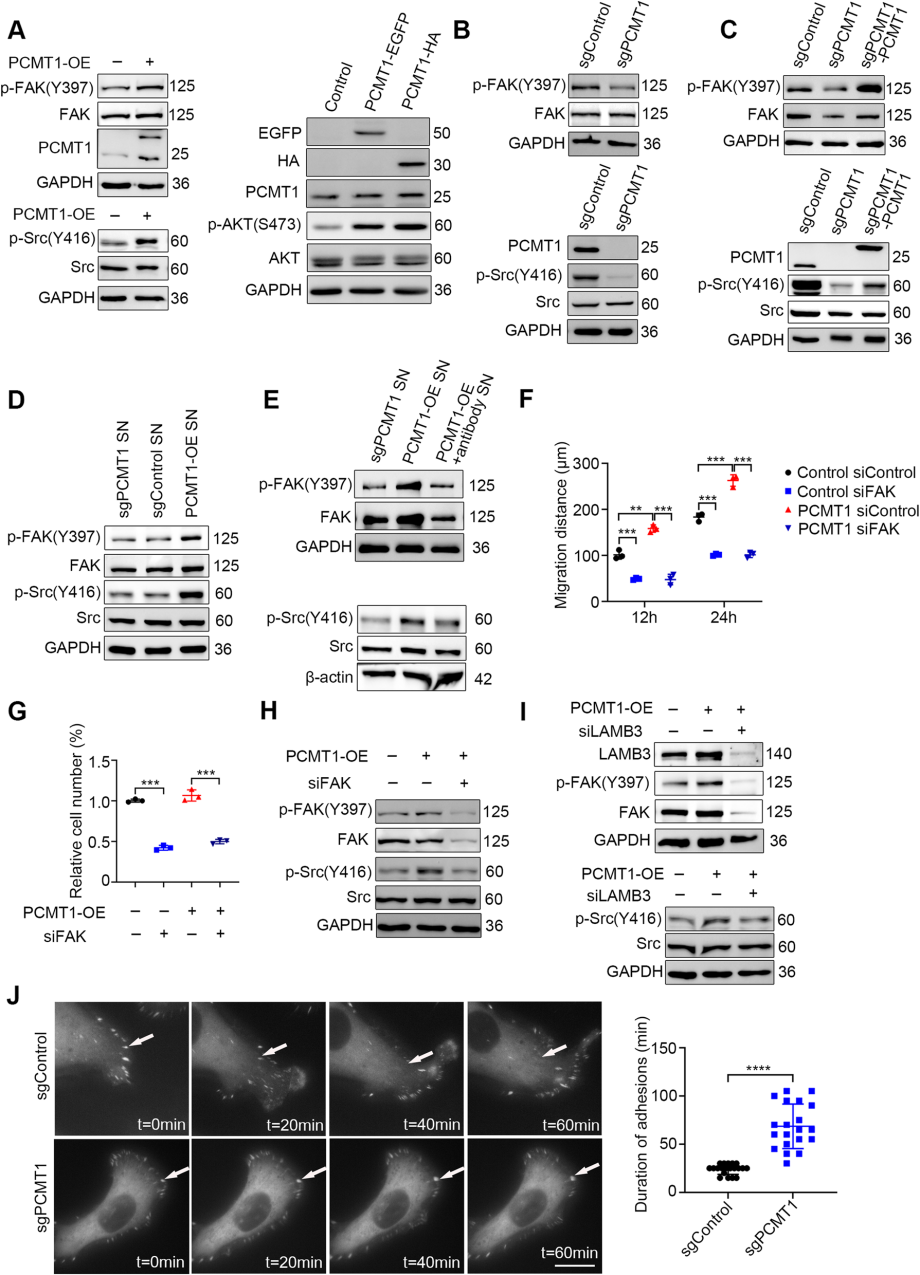
PCMT1 regulates the integrin-FAK-Src pathway through LAMB3. **A** and **B** Western blot analysis of the phosphorylation of FAK-Src and AKT in PCMT1-OE SKOV3 cells (**A**) and *PCMT1*-KO SKOV3 cells (**B**). **C** The impact of rescue expression of PCMT1 on FAK-Src signaling in *PCMT1*-KO SKOV3 cells was also determined. **D** *PCMT1*-KO cells were incubated with the supernatant (SN) from PCMT1-OE SKOV3 cells, and FAK-Src signaling was determined. **E** The effect of SN incubated with PCMT1 blocking antibody on FAK-Src was also examined under the same conditions. **F–H** The effects of *FAK* silencing on cell migration (**F**) and living cell spheroid formation (**G**) along with FAK-Src phosphorylation (**H**) were detected in PCMT1-OE SKOV3 cells. **I** After silencing *LAMB3* in PCMT1-OE SKOV3 cells, FAK-Src signal activity was determined. **J** Control (sgControl) and *PCMT1*-KO (sgPCMT1) SKOV3 cells were transfected with EGFP-tagged paxillin, and cell FA dynamics were monitored and photographed every 5 min. Representative frames from time-lapse sequences (left) and quantification of duration time are shown (right, *n* = 20). (scale bar: 15 μm; Data are shown as mean ± SEM of 3 independent experiments. Data were statistically analyzed with Student's t-test. *P < 0.05; **P < 0.01; ***P < 0.001.)

According to our IP-MS results, the mechanism by which PCMT1 promotes cell invasiveness was investigated in the context of ECM-epithelial cell interactions. We focused on the FAK-Src pathway, as it has been implicated in integrin-focal adhesion-dependent signals. Upon integrin activation, FAK is activated by autophosphorylation of Tyr397 and recruits Src, PI3-kinase, paxillin and other focal adhesion substrates. Notably, PCMT1 overexpression resulted in activation of FAK due to phosphorylation of the Tyr397 site as well as marked activation of phosphorylation of Src Tyr416 and phosphorylation of Akt Ser473 in SKOV3 cells (Fig. 6A). In contrast, knockout of PCMT1 reduced the levels of FAK phosphorylation and Src phosphorylation without changing their total protein levels (Fig. 6B). Importantly, introduction of PCMT1-WT-HA into *PCMT1-KO* cells resulted in restoration of FAK and Src phosphorylation, suggesting that PCMT1 is required for activation of the FAK-Src pathway (Fig. 6C).

Considering our finding that extracellular PCMT1 plays roles in cancer cell invasiveness, we evaluated whether secreted PCMT1 affects the FAK-Src pathway. We found that the phosphorylation of FAK and Src was increased in *PCMT1*-KO cells upon treatment with supernatant from cells overexpressing PCMT1 (Fig. 6D). More importantly, this effect was abolished if the supernatant from PCMT1-OE cells was pretreated with PCMT1 blocking antibody. As shown in Fig. 6E, FAK pTyr397 and Src pTyr416 were increased when *PCMT1*-KO SKOV3 cells were cultured with PCMT1-OE cell supernatant and were diminished when the supernatant was pretreated with PCMT1 blocking antibody (Fig. 6E).

To determine whether PCMT1 promotes cell migratory capacity and invasiveness through integrin-FAK-Src, we knocked down *FAK* in control and PCMT1-OE SKOV3 cells, and examined the cell migration and invasion potentials. The results showed that upon silencing *FAK*, PCMT1-enhanced cell migration was abolished (Fig. 6F and Fig. S7A). Moreover, the number of viable cells in the spheroid formation assay was markedly reduced by FAK deficiency in both control and PCMT1-OE cells under ULA culture conditions (Fig. 6G). These results suggest that activation of the FAK signaling pathway was at least partially essential for PCMT1-induced anoikis resistance and migration potential. Consistent with previous results, the levels of Src phosphorylation were increased after overexpressing PCMT1. Silencing of *FAK* in PCMT1-OE cells reduced the levels of Src phosphorylation without changing the total Src level (Fig. 6H). As we found that PCMT1 interacted with LAMB3, the extracellular ligand of integrin, we examined whether PCMT1 regulates the FAK-Src pathway through LAMB3. In PCMT1-OE SKOV3 cells, we found that knockdown of *LAMB3* diminished PCMT1-induced FAK-Src activation, as determined by the levels of FAK pY397 and Src pY416 (Fig. 6I), further supporting the result that silencing *LAMB3* inhibits cell migration (Fig. 4F). These findings collectively demonstrate that FAK and LAMB3 are required in PCMT1-induced cell migration and spheroid formation.

In light of the above results, we speculated that PCMT1 may influence cell focal adhesion (FA) dynamics and consequently affect cell motility. We transfected EGFP-tagged paxillin, an FA marker, into control (sgControl) and *PCMT1*-KO (sgPCMT1) cells and performed long-term live-cell imaging to explore what effect PCMT1 has on cell focal adhesion dynamics by monitoring the paxillin-EGFP signal. FA turnover is activated in motile cells, leading to less stable adhesions. We observed that the duration of adhesions was longer in the *PCMT1*-KO group than in the control group (20 cells *vs* 20 cells, *p* < 0.0001) (Fig. 6J). In line with this finding, *PCMT1*-KO cells (Supplementary video 1) displayed little motility on glass compared with control cells (Supplementary video 2). These data indicated that PCMT1 plays an important role in maintaining focal adhesion dynamics, especially disassembly and cell motility.

### PCMT1 promotes ovarian cancer cell metastasis in vivo

**Fig. 7:**
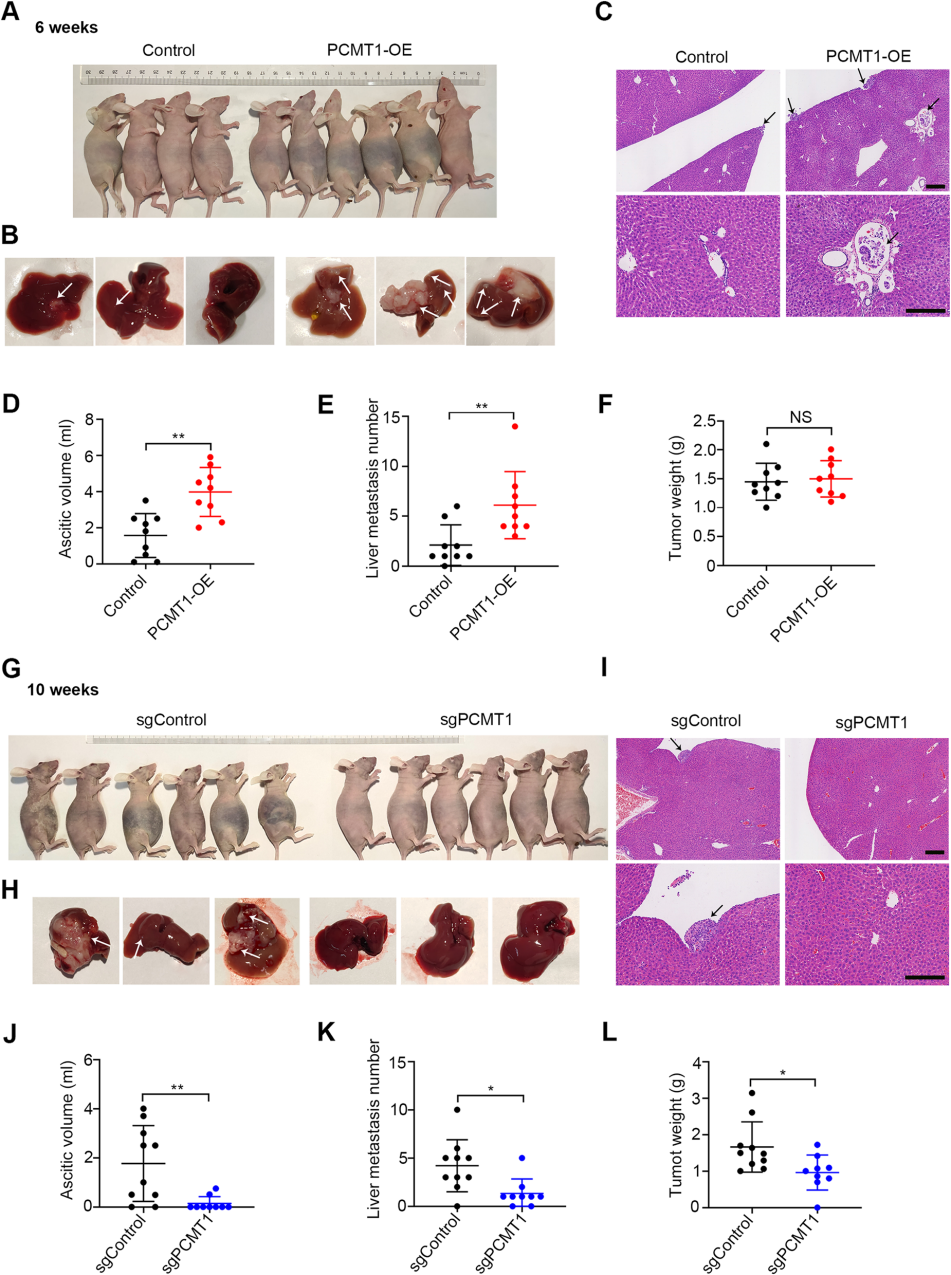
PCMT1 promotes ovarian cancer cell metastasis in vivo. **A-E** Female BALB/c nude mice were injected intraperitoneally with control or PCMT1-OE SKOV3 cells. After 6 weeks, the mice were anesthetized and photographed (**A**). The livers were dissected and photographed (**B**) and subjected to H&E staining (**C**). The ascitic volume (**D**), the number of metastatic lesions in the liver (**E**) and the total tumor weight (**F**) were examined (*n* = 9). **G-L** Female BALB/c nude mice were injected intraperitoneally with control (sgControl) or *PCMT1*-KO (sgPCMT1) SKOV3 cells. After 10 weeks, mice were photographed (**G**), and livers were dissected (**H**) for H&E staining analysis. The tumor tissue is highlighted by arrow (**I**). The ascitic volume (**J**), the number of metastatic lesions in the liver (**K**) and the total tumor weight (**L**) were also determined between the control group (*n* = 10) and the *PCMT1* knockout group (*n* = 9). Each dot represents one animal (scale bar: 200 μm; Data were statistically analyzed with Student's t-test. *P < 0.05; **P < 0.01; ***P < 0.001.)

Because clinical data indicated that high PCMT1 expression correlated with late stage and poor survival, we examined whether PCMT1 overexpression or knockout in SKOV3 cells can affect their ability to form metastasis in an in vivo intraperitoneal (i.p.) injection model, as described previously [30]. Female BALB/c nude mice were randomly divided into four groups and injected with different SKOV3 stable transfectants: PCMT1 stable overexpressing cells (SKOV3-PCMT1-OE) and stable luc overexpression cells as a control (SKOV3-control), and *PCMT1* knockout cells (sgPCMT1) and their matched controls (sgControl). Approximately six weeks after injection, mice injected with PCMT1-OE and control cells were sacrificed since SKOV3-PCMT1-OE cells promoted remarkable ascites formation (Fig. 7A). The ascites volume of mice bearing SKOV3-PCMT1-OE tumors was significantly larger than that of the SKOV3-control group mice (Fig. 7D). In line with this finding, metastases were more frequent in mice injected with SKOV3-PCMT1-OE cells, especially in the peritoneum, mesentery (Fig. S8A), diaphragm, spleen (Fig. S8B) and liver. We found that 9 of 10 mice injected with PCMT1-OE cells exhibited liver metastasis, and the frequency and number of metastases were higher in the PCMT1-OE group than in the control group (Fig. 7B and E, P = 0.0076). Interestingly, H&E-stained sections of liver tissue showed that tumor cells infiltrated the blood vessels in the SKOV3-PCMT1-OE group but not in the control group (Fig. 7C). This suggested that PCMT1 could promote cancer cell spreading to other organs through blood vessels. There was no difference in tumor weight between the two groups (Fig. 7F), indicating that overexpression of PCMT1 mainly enhanced the metastatic ability of tumor cells in vivo. In contrast, tumor growth and ascites formation in the *PCMT1* knockout group progressed much slower than those in the control group. Ten weeks after tumor implantation, we sacrificed the mice and measured ascites volume, tumor weight and metastasis number. The ascites volume of mice bearing *PCMT1* knockout (sgPCMT1) tumors was significantly smaller than that of the control (sgControl) group (Fig. 7G and J, P = 0.0063). In addition, mice injected with sgControl cells also developed metastatic tumors in the peritoneum, mesentery, diaphragm, and liver. However, PCMT1 knockout cells only developed small tumors with less organ involvement in the abdominal cavity (Fig. S8C). Compared to that in the control group, the number of metastases in the liver was significantly decreased in the sgPCMT1 group (Fig. 7H and K, P = 0.012). Histological analysis of the xenografts revealed that tumors from the control groups invaded into the liver, while such invasive behavior was reduced in the *PCMT1* knockout group (Fig. 7I). Moreover, the tumor weight of the *PCMT1* knockout group was significantly lighter than that of the control group (Fig. 7 L). Collectively, these in vivo observations suggested that PCMT1 is a critical factor for tumor growth, ascites formation and metastasis in EOC.

## Discussion

Metastatic potential and anoikis resistance are the main reasons for the poor prognosis of EOC. However, the mechanism underlying these biological processes remain elusive. In this study, we present the first comprehensive screening for the genes that may participate in ovarian cancer metastasis and anoikis resistance using a well-designed whole genome CRISPR/Cas9 screen and identified the methyltransferase PCMT1 as an important driver in metastasis formation and anoikis resistance. We demonstrated that through LAMB3, PCMT1 activates integrin-FAK-Src signaling to enhance cancer cell adhesion, migration, and invasion and promote metastasis formation. Further, its invasion potential can be inhibited by a PCMT1 blocking antibody, and patient data sets point to a positive correlation between PCMT1 expression and ovarian cancer metastasis.

It has been reported that increased deposition of ECM proteins, such as laminin 332, fibronectin and collagens, provides necessary biochemical and mechanical cues to promote cancer cell migration and invasion [31]. *LAMB3* encodes one of the three subunits of laminin-332 (LM-332), an epithelial-basal lamina-specific LM variant composed of the α3, β3 and γ2 chains. It has been reported that LAMB3 is involved in the invasive and metastatic abilities of some types of cancer, such as colon, pancreas, lung, cervix, stomach, and prostate cancer [32–34]. Currently, multiple regulation models of laminin 332 have been discovered in tumor progression, including transcription and protein stabilization modulation [35, 36]. In this study, PCMT1 was identified to bind with LAMB3 and affect the phosphorylation level of FAK-Src, the downstream signal of the α3β1 integrin receptor, suggesting that PCMT1 appears to act via the laminin 332/α3β1-integrin complex. PCMT1 is likely to affect LAMB3 via a posttranslational modification manner, as indicated by the altered protein amount and constant mRNA level. It is interesting to note that when ovarian cancer cells are detached from the ECM, upregulation of both PCMT1 and LAMB3 and spheroid formation are observed (Fig. 3G). We therefore reasoned that PCMT1 exerts a transient action rather than a long-term impact. Considering the biochemical characteristics of PCMT1 as a protein repair enzyme, it was likely that PCMT1 interacts with and repairs LAMB3 to protect its function and stability, which would promote the laminin 332/α3β1 integrin receptor-mediated FAK-Src pathway. These findings identify PCMT1 as a new regulatory molecule implicated in the laminin 332/α3β1 integrin signal cascade and extend the concept, which emphasizes the important role of laminin 332/α3β1 integrin signaling in cancer metastasis. Further study will be needed to elucidate the detailed mechanism underlying PCMT1/LAMB3 interation and dissect the regulatory mechanism that drives the changes in PCMT1 levels during the process of cancer cell detachment.

Previous studies and databases have reported that PCMT1 expression is positively correlated with poor prognosis in several human cancers, including breast cancer, bladder cancer, and endometrial cancer [15, 37]. However, the exact role of PCMT1 in cancer progression is not clear. In this study, we evaluated the clinical relevance of PCMT1 levels with ovarian cancer progression in patient samples and dissected the mechanism of how extracellular PCMT1 contributes to cancer progression through modulation of LAMB3, which may be used as a serum marker for monitoring metastasis. More importantly, since the antibody blocking PCMT1 significantly inhibited cancer cell migration and invasion, this finding may provide a novel strategy for therapeutic intervention of metastatic ovarian cancer with PCMT1-targeting antibodies or inhibitors. To further survey the effect of PCMT1-targeting antibody toward its clinical application, further investigation of its precise targeting ability was needed.

Moreover, we noticed a significant cluster of genes involved in protein repair (Fig. S1D) in the enrichment analysis of the CRISPR/Cas9 screen. This suggests that there are more proteins participating in the protein repair process during cancer cell detachment, and thus, the protein repair mechanism may play an important role during the acquisition of metastatic capacity.

PCMT1 is localized to multiple regions, including the cytosol and extracellular space, and it may also be present in exosomes and vesicles [38, 39]. We found PCMT1 to be a secreted protein targeting extracellular LAMB3 without encapsulation, implying diverse secretory forms of PCMT1 in biological processes. PCMT1 has been reported to be involved in apoptosis through several different manners. PCMT1 can interact and negatively regulate the tumor suppressor protein p53 (reduced protein level and activity) by carboxyl methylation of p53 at isoaspartate residues 29 and 30, which in turn represses apoptosis and growth arrest and contributes to cancer progression [40]. In dopamine-induced neuroblastoma cell death, PCMT1 protects cells from apoptosis by affecting ROS levels and caspase-3/9 activity [41]. PCMT1 also inhibits apoptosis caused by Bax overexpression [13]. Moreover, MST1 is a target of PCMT1 that ameliorates neuronal apoptosis by inhibiting MST1 activation [42]. Conversely, downregulation of PCMT1 increases apoptosis upon DNA damage in leukemia [43]. In line with that, our data demonstrated that knockout of *PCMT1* in the anoikis-resistant SKOV3 ovarian carcinoma cell line caused significant apoptosis in response to detachment from the ECM. This change was associated with strong inhibition of tumorigenicity and metastasis of the *PCMT1*-knockout SKOV3 cells in the i.p. xenograft model. Taken together, the above findings suggest that PCMT1 can impact apoptosis in a variety of ways and indicate the important concept that PCMT1 could be a crucial molecule in cancer progression.

This study has some limitations. First, the term ovarian cancer describes a highly heterogeneous group of tumors which show distinct cellular origin, genetic aberrations and prognosis in patients. However, in this study, we mainly examined the expression of PCMT1 in serous ovarian cancer with limited number. Hence, for comprehensive study the role of PCMT1 in ovarian cancer progression, large cohort of patient samples with different histological type of ovarian cancer was needed. Cancer cell models such as patient derived cancer cells will be greatly helpful for further mechanism dissection. Moreover, given the finding that PCMT1 was secreted into the ECM and its role in catalyzing the methyl esterification of L-isoaspartyl and D-aspartyl residues in proteins, it may act as a key regulator in maintaining the function of extracellular matrix proteins. Therefore, comprehensive analysis of the global change in the deamidation/isomerization status of all proteins, especially in the extracellular space during cancer metastasis, by proteomics analysis may help us to better understand the role of PCMT1 in promoting this process.

## Conclusions

In conclusion, in the first systematic study to identify anoikis resistance drivers, we found that PCMT1 enhances cell adhesion, migration and invasion, upregulates prometastatic FAK-Src signaling, and increases in vivo metastasis formation and that its expression is positively correlated with metastatic stage in human ovarian cancer. Moreover, PCMT1 is secreted from cancer cells to the extracellular space, suggesting that serum PCMT1 levels may serve as a promising marker of the metastatic propensity of ovarian cancer. Importantly, in vitro intervention with an antibody targeting PCMT1 almost abolished the effect of secreted PCMT1, illustrating a potential medical application for ovarian cancer metastasis intervention.

## Supplementary Information

**Additional file 1: Figure S1. **A genome-wide CRISPR/Cas9 screen identified that PCMT1 is important in ovarian cancer progression. (A) Schematic diagram of the generation of SKOV3-GeCKO. (B) Western blot analysis of cas9 expression in sgRNA library cells. (C) The top fold change genes of negative and positive screen were showed. (D) GO enrichment pathways of the top 1000 genes (negative screen) analyzed by the RIGER method. (E) qRT-PCR analysis of candidate gene expression between primary serous ovarian cancer and metastatic tumors of 4 patients. (F) qRT-PCR analysis of *KCTD10* (upper) and *ACTR10* (lower) expression in 14 pairs of in situ ovarian cancer tissues and metastatic cancer tissues. (*P < 0.05; **P < 0.01; ***P < 0.001.).** Figure S2.** PCMT1 deletion inhibits metastasis-relevant traits *in vitro*. (A) Representative images of the cell adhesion assay (left) and analyses of adhesion capacity (right) comparing control SKOV3 and *PCMT1* knockout SKOV3 cells (using Fibronectin) (B) Western blot (left) and qRT-PCR (right) analyses verified the knockdown efficiency in OVCAR3 cells. (C) Representative images of cell spheroids in OVCAR3 cells after knocking down *PCMT1.* (D) After ULA cultured for 72h, the amounts of apoptotic-antiapoptotic proteins in PCMT1 knockout SKOV3 cells were determined by western blot. (E) Representative images and quantification of migrated cells cultured for 24 h or 48 h in the control group and *PCMT1* knockdown group in Hela cells. (F) Western blot analyses verified the* PCMT1* knockdown efficiency in Hela cells. (G) Representative images and quantification of migrated cells cultured for 48 h in the control group and* PCMT1* knockdown group in Hela cells by transwell assay. (scale bar: 200 μm; Data are shown as mean ± SEM of 3 independent experiments. *P < 0.05; **P < 0.01; ***P < 0.001.).** Figure S3.** Re-expression of PCMT1 in knockout cells promotes traits metastasis-relevant traits *in vitro*. (A) and (B) Representative images of the cell adhesion assay (Up: Laminin; down: Fibronectin) and analyses of adhesion capacity comparing control and *PCMT1 *knockdown Hela cells (Left: Laminin; right: Fibronectin) (C) Western blot analysis of PCMT1 in control and *PCMT1*-knockout and PCMT1-reexpressing SKOV3 cells. (D) Representative images of migrated cells cultured for 12 h or 24 h in the control group,* PCMT1*-knockout group and PCMT1-reexpressing group. (E) Representative images of SKOV3 cell adhesion in control and *PCMT1*-knockout and PCMT1-reexpressing SKOV3 cells. (F) Cell proliferation was tested in the control, *PCMT1*-knockout and PCMT1-reexpressing groups (scale bar: 200 μm; Data are shown as mean ± SEM of 3 independent experiments. *P < 0.05; **P < 0.01; ***P < 0.001.).** Figure S4. **PCMT1 promotes cell adhesion in OVCAR3 cells. (A) OVCAR3 control cells, EGFP-tagged PCMT1-OE cells and HA-tagged PCMT1 cells were examined by western blotting using an antibody against PCMT1. (B) Representative immunofluorescence images of the subcellular localization of PCMT1 in OVCAR3 cells. (C) The cell proliferation of control cells and PCMT1-OE OVCAR3 cells was monitored for 5 days by CCK-8 assay. (D) Left: The number of living cells was measured in spheroid formation for 5 days in the above two cell types; Right: Western blot analysis of PCMT1 in OVCAR3 and SKOV3 cells. (E) The assessment of cell adhesion in control or PCMT1-OE cells under Laminin/fibronectin-coated conditions after 30 min of attachment (scale bar: 25 μm; Data are shown as mean ± SEM of 3 independent experiments. *P < 0.05; **P < 0.01; ***P < 0.001.).** Figure S5. **IP-MS identified the proteins that interact with PCMT1. (A) The overlap of four data sets: control (Luc) vs PCMT1; non-crosslinking vs crosslinking (DTBP) derived from IP-MS analysis. (B) Reconstruction of the protein-protein interaction network of the 39 proteins in the intersection of PCMT1 and PCMT1+DTBP data sets was used for analysis based on the STRING database. The interactions are shown in the form of network. Thicker lines represent a stronger association. The proteins are identified by their gene names located near each sphere. (C) GO term enrichment of interacting with the PCMT1 protein set. (D) Representative immunofluorescence images of the subcellular localization of PCMT1 and LAMB3 in SKOV3 cells.** Figure S6. **Supernatant from PCMT1-OE cells does not affect cell migration. (A) *PCMT1*-KO (sgPCMT1) SKOV3 cells were incubated with supernatant derived from control (sgControl) cells or PCMT1-OE cells for 24 h and 48 h. Cell migration was examined and quantified.** Figure S7. ***FAK* knockdown in PCMT1-OE cells inhibits cell migration. (A) PCMT1-OE SKOV3 cells were transfected with control siRNA and FAK siRNA, and cell migration was assessed.** Figure S8. **PCMT1 promotes ovarian cancer cell metastasis *in vivo*. (A and B) Representative images of metastatic tumors in the mesenterium (A) and spleen (B) in mice injected with control or PCMT1-OE SKOV3 cells. (C) Representative images of metastatic tumors in the mesenterium in mice injected with control (sgControl) or *PCMT1*-KO (sgPCMT1) SKOV3 cells.

**Additional file 2:** **Table S1.** The siRNAs used for specific genes knockdown. **Table S2.** Primers used for real-time PCR amplification**. Table S3. **The sgRNA used for specific genes knockout..

**Additional file 3.**

**Additional file 4.**

## Data Availability

The datasets used and/or analyzed during the current study are available from the corresponding author [fww12066@rjh.com.cn] upon reasonable request.

## Abbreviations

ECM: Extracellular matrix
PCMT1: Protein-L-isoaspartate (D-aspartate) O-methyltransferase
FAK: Focal adhesion kinase
ILK: Integrin-linked kinase
MAPK: Mitogen-activated protein kinase
FBS: Fetal bovine serum
MCLB: Mammalian cell lysis buffer
ECL: Enhanced chemiluminescence
IP-MS: Immunoprecipitation followed by mass spectrometry
DTBP: Dimethyl dithiobispropionimidate
luc: Luciferase
NGS: Next-generation sequencing
RIGER: RNAi Gene Enrichment Ranking
PEI: Polyethylenimine
qRT-PCR: Quantitative real-time PCR
IHC: Immunohistochemistry
CCK-8: Cell Counting Kit-8
OD: Optical density
DAPI: 4,6-Diamidino-2-phenylindole
ORF: Open-reading frame
UTR: Untranslated region
IVT: In vitro Transcription
ARCA: Anti-reverse cap analog
MOI: Multiplicity of infection
ULA: Ultralow-attachment
GO: Gene Ontology
EOC: Epithelial ovarian cancer
TCGA: The Cancer Genome Atlas
OS: Overall survival
PFS: Progression-free survival
KO: Knockout
FN: Fibronectin
OE: Overexpressing
STRING: Search Tool for the Retrieval of Interacting Genes/Proteins
KEGG: Kyoto Encyclopedia of Genes and Genomes
FA: Focal adhesion
H&E: Hematoxylin and eosin
PBS: Phosphate buffered solution
