# Supplementary material for: Genome-wide CRISPR/Cas9 library screen identifies PCMT1 as a critical driver of ovarian cancer metastasis
Source: J Exp Clin Cancer Res. 2022 Jan 15;41:24. doi: 10.1186/s13046-022-02242-3 (PMC8760697; doi:10.1186/s13046-022-02242-3)
Supplement: Supplementary file 1 — Additional file 1: Figure S1. A genome-wide CRISPR/Cas9 screen identified that PCMT1 is important in ovarian cancer progression. (A) Schematic diagram of the generation of SKOV3-GeCKO. (B) Western blot analysis of cas9 expression in sgRNA library cells. (C) The top fold change genes of negative and positive screen were showed. (D) GO enrichment pathways of the top 1000 genes (negative screen) analyzed by the RIGER method. (E) qRT-PCR analysis of candidate gene expression between primary serous ovarian cancer and metastatic tumors of 4 patients. (F) qRT-PCR analysis of KCTD10 (upper) and ACTR10 (lower) expression in 14 pairs of in situ ovarian cancer tissues and metastatic cancer tissues. (*P < 0.05; **P < 0.01; ***P < 0.001.). Figure S2. PCMT1 deletion inhibits metastasis-relevant traits in vitro. (A) Representative images of the cell adhesion assay (left) and analyses of adhesion capacity (right) comparing control SKOV3 and PCMT1 knockout SKOV3 cells (using Fibronectin) (B) Western blot (left) and qRT-PCR (right) analyses verified the knockdown efficiency in OVCAR3 cells. (C) Representative images of cell spheroids in OVCAR3 cells after knocking down PCMT1. (D) After ULA cultured for 72h, the amounts of apoptotic-antiapoptotic proteins in PCMT1 knockout SKOV3 cells were determined by western blot. (E) Representative images and quantification of migrated cells cultured for 24 h or 48 h in the control group and PCMT1 knockdown group in Hela cells. (F) Western blot analyses verified the PCMT1 knockdown efficiency in Hela cells. (G) Representative images and quantification of migrated cells cultured for 48 h in the control group and PCMT1 knockdown group in Hela cells by transwell assay. (scale bar: 200 μm; Data are shown as mean ± SEM of 3 independent experiments. *P < 0.05; **P < 0.01; ***P < 0.001.). Figure S3. Re-expression of PCMT1 in knockout cells promotes traits metastasis-relevant traits in vitro. (A) and (B) Representative images of the cell adhesion assay (U [file 13046_2022_2242_MOESM1_ESM.docx]

**Supplementary Figure and figure legends**

**Figure S1**


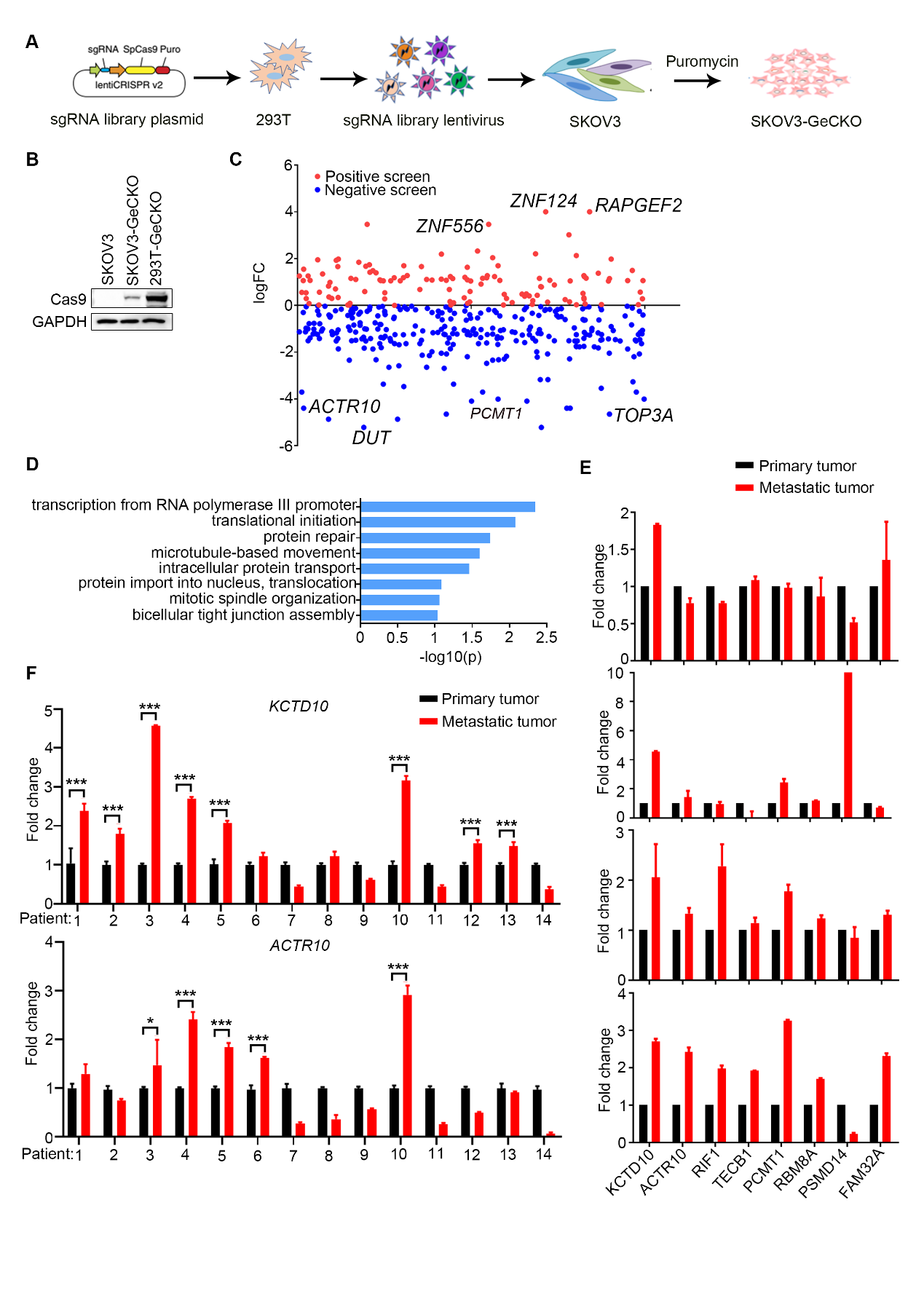


**Figure S1 A genome-wide CRISPR/Cas9 screen identified that PCMT1 is important in ovarian cancer progression.**

(A) Schematic diagram of the generation of SKOV3-GeCKO. (B) Western blot analysis of cas9 expression in sgRNA library cells. (C) The top fold change genes of negative and positive screen were showed. (D) GO enrichment pathways of the top 1000 genes (negative screen) analyzed by the RIGER method. (E) qRT-PCR analysis of candidate gene expression between primary serous ovarian cancer and metastatic tumors of 4 patients. (F) qRT-PCR analysis of *KCTD10* (upper) and *ACTR10* (lower) expression in 14 pairs of in situ ovarian cancer tissues and metastatic cancer tissues. (*P < 0.05; **P < 0.01; ***P < 0.001.)

**Figure S2**


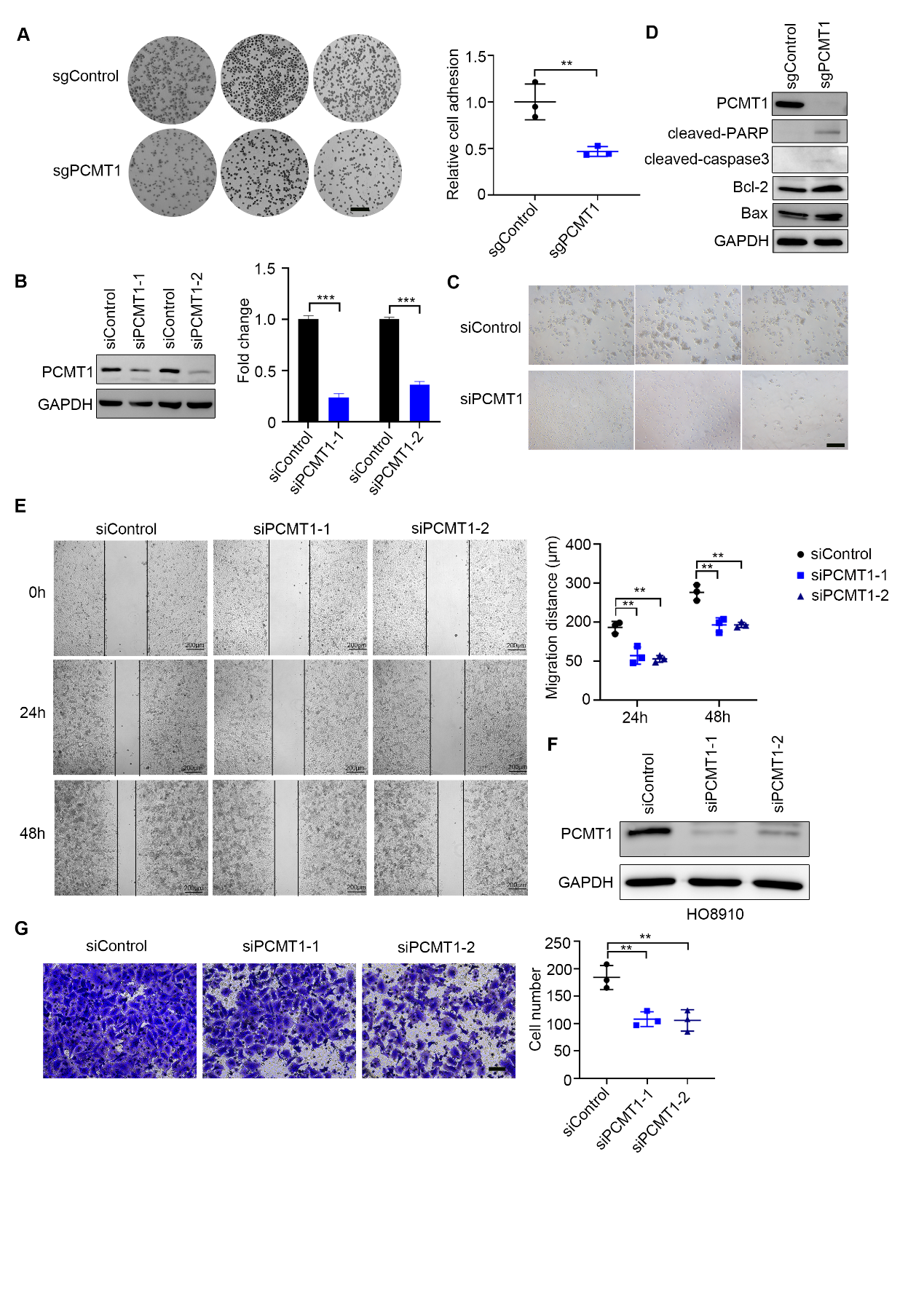


**Figure S2 PCMT1 deletion inhibits metastasis-relevant traits *in vitro*.**

(A) Representative images of the cell adhesion assay (left) and analyses of adhesion capacity (right) comparing control SKOV3 and *PCMT1* knockout SKOV3 cells (using Fibronectin) (B) Western blot (left) and qRT-PCR (right) analyses verified the knockdown efficiency in OVCAR3 cells. (C) Representative images of cell spheroids in OVCAR3 cells after knocking down *PCMT1.* (D) After ULA cultured for 72h, the amounts of apoptotic-antiapoptotic proteins in PCMT1 knockout SKOV3 cells were determined by western blot. (E) Representative images and quantification of migrated cells cultured for 24 h or 48 h in the control group and *PCMT1* knockdown group in Hela cells. (F) Western blot analyses verified the *PCMT1* knockdown efficiency in Hela cells. (G) Representative images and quantification of migrated cells cultured for 48 h in the control group and *PCMT1* knockdown group in Hela cells by transwell assay. (scale bar: 200 μm; Data are shown as mean ± SEM of 3 independent experiments. *P < 0.05; **P < 0.01; ***P < 0.001.)

**Figure S3**


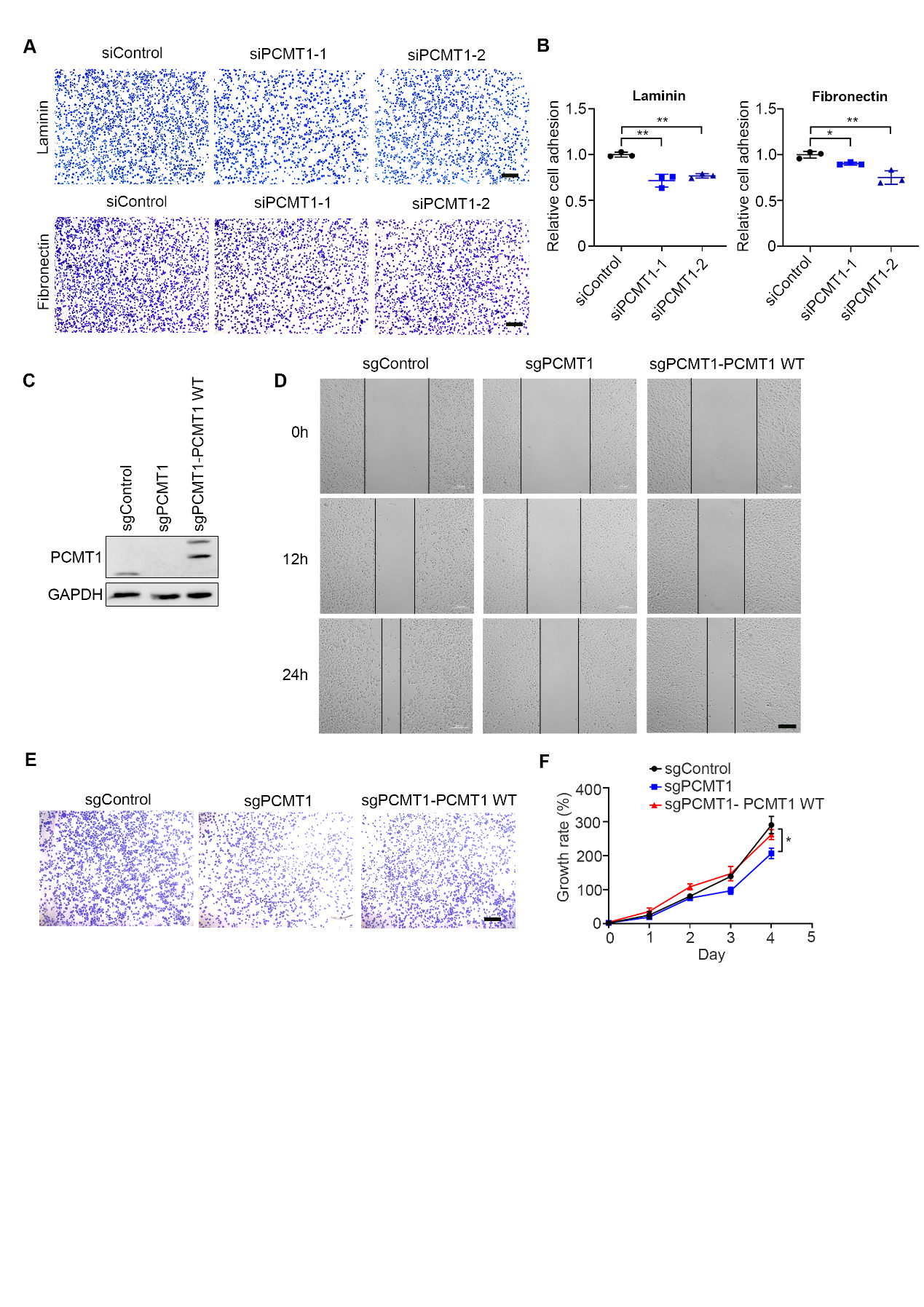


**Figure S3 Re-expression of PCMT1 in knockout cells promotes traits metastasis-relevant traits *in vitro*.**

(A) and (B) Representative images of the cell adhesion assay (Up: Laminin; down: Fibronectin) and analyses of adhesion capacity comparing control and *PCMT1* knockdown Hela cells (Left: Laminin; right: Fibronectin) (C) Western blot analysis of PCMT1 in control and *PCMT1*-knockout and PCMT1-reexpressing SKOV3 cells. (D) Representative images of migrated cells cultured for 12 h or 24 h in the control group, *PCMT1*-knockout group and PCMT1-reexpressing group. (E) Representative images of SKOV3 cell adhesion in control and *PCMT1*-knockout and PCMT1-reexpressing SKOV3 cells. (F) Cell proliferation was tested in the control, *PCMT1*-knockout and PCMT1-reexpressing groups (scale bar: 200 μm; Data are shown as mean ± SEM of 3 independent experiments. *P < 0.05; **P < 0.01; ***P < 0.001.)

**Figure S4**


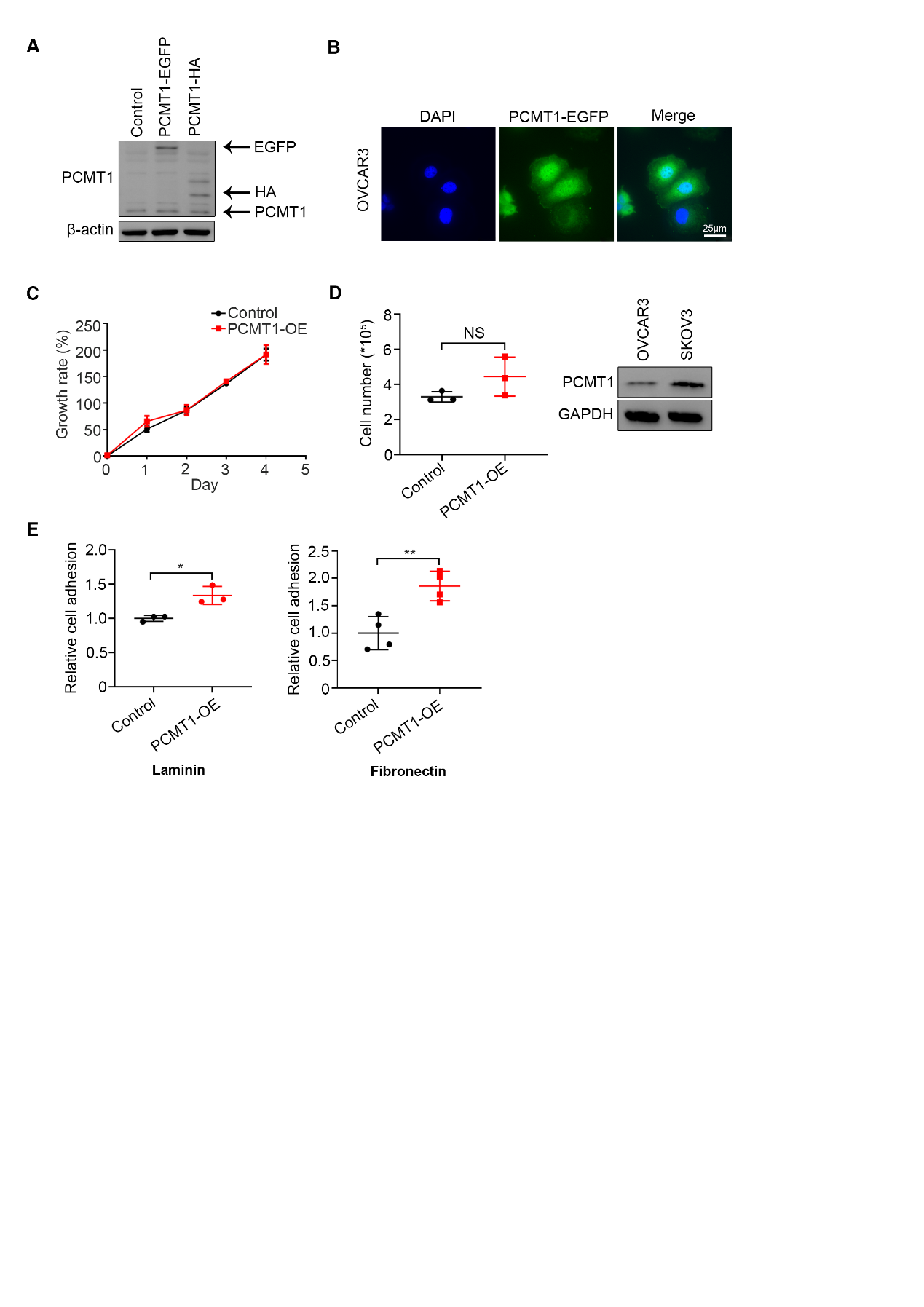


**Figure S4 PCMT1 promotes cell adhesion in OVCAR3 cells.**

(A) OVCAR3 control cells, EGFP-tagged PCMT1-OE cells and HA-tagged PCMT1 cells were examined by western blotting using an antibody against PCMT1. (B) Representative immunofluorescence images of the subcellular localization of PCMT1 in OVCAR3 cells. (C) The cell proliferation of control cells and PCMT1-OE OVCAR3 cells was monitored for 5 days by CCK-8 assay. (D) Left: The number of living cells was measured in spheroid formation for 5 days in the above two cell types; Right: Western blot analysis of PCMT1 in OVCAR3 and SKOV3 cells. (E) The assessment of cell adhesion in control or PCMT1-OE cells under Laminin/fibronectin-coated conditions after 30 min of attachment (scale bar: 25 μm; Data are shown as mean ± SEM of 3 independent experiments. *P < 0.05; **P < 0.01; ***P < 0.001.)

**Figure S5**


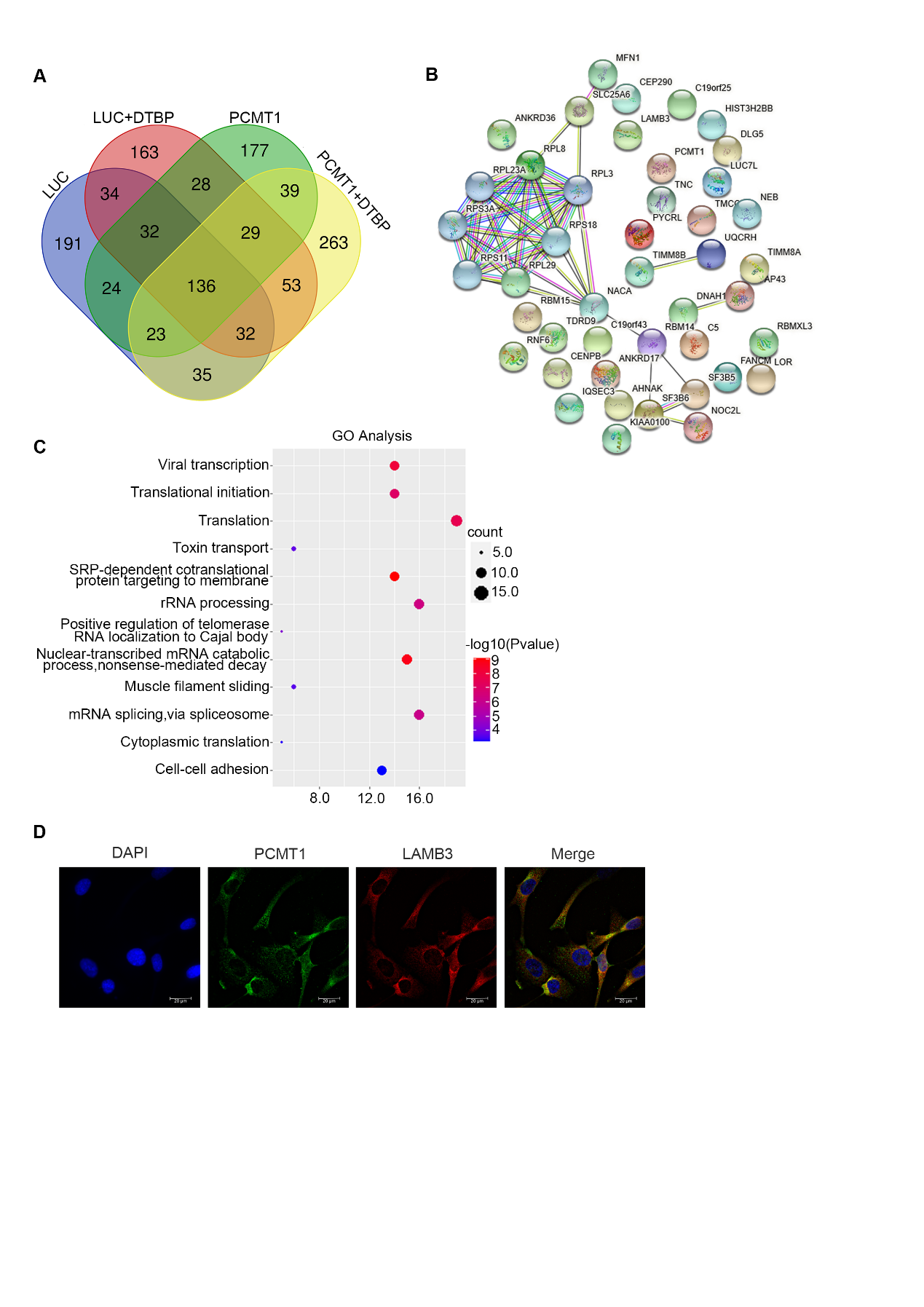


**Figure S5 IP-MS identified the proteins that interact with PCMT1.** (A) The overlap of four data sets: control (Luc) vs PCMT1; non-crosslinking vs crosslinking (DTBP) derived from IP-MS analysis. (B) Reconstruction of the protein-protein interaction network of the 39 proteins in the intersection of PCMT1 and PCMT1+DTBP data sets was used for analysis based on the STRING database. The interactions are shown in the form of network. Thicker lines represent a stronger association. The proteins are identified by their gene names located near each sphere. (C) GO term enrichment of interacting with the PCMT1 protein set. (D) Representative immunofluorescence images of the subcellular localization of PCMT1 and LAMB3 in SKOV3 cells.

**Figure S6**


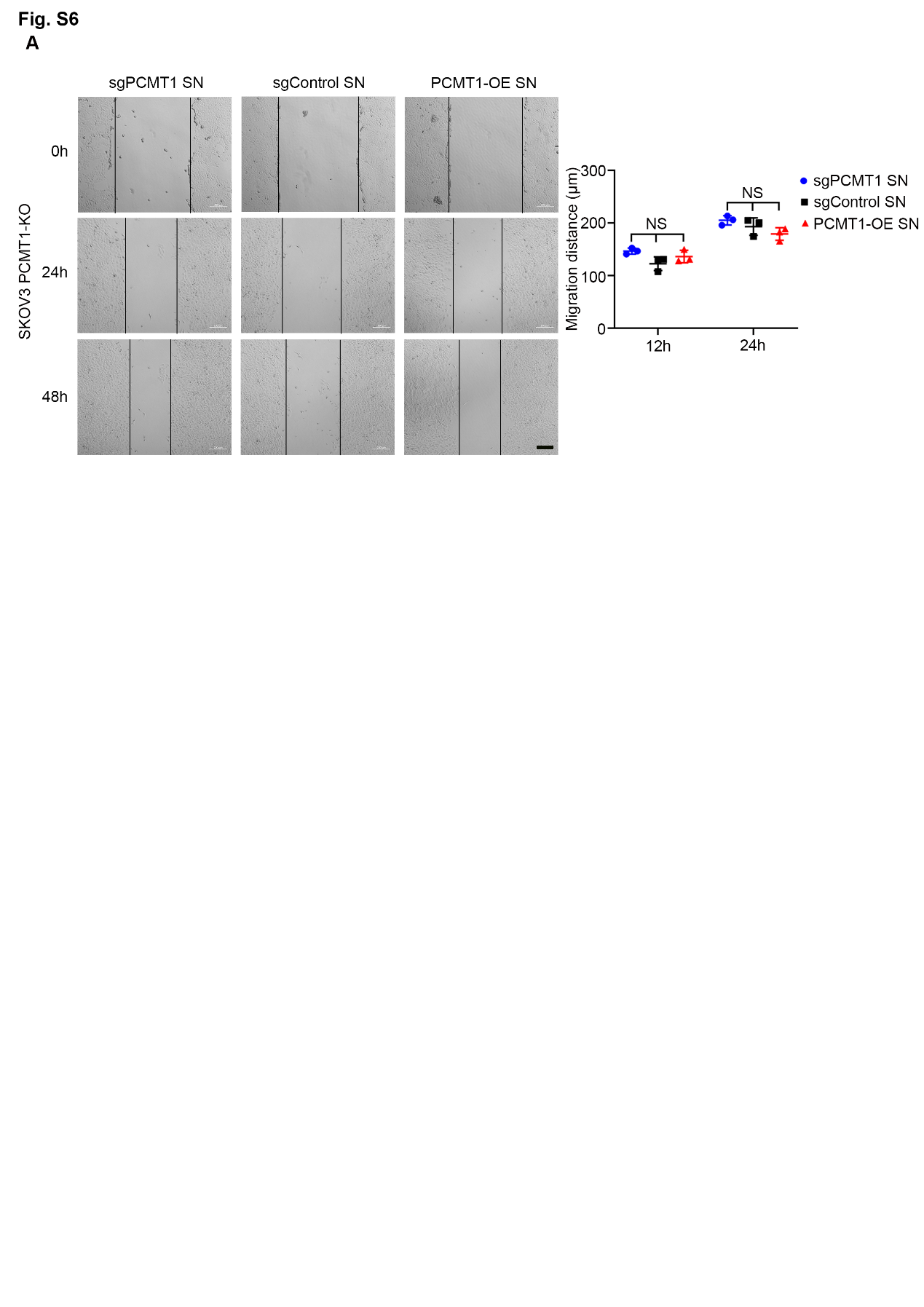


**Figure S6 Supernatant from PCMT1-OE cells does not affect cell migration.** (A) *PCMT1*-KO (sgPCMT1) SKOV3 cells were incubated with supernatant derived from control (sgControl) cells or PCMT1-OE cells for 24 h and 48 h. Cell migration was examined and quantified.

**Figure S7**


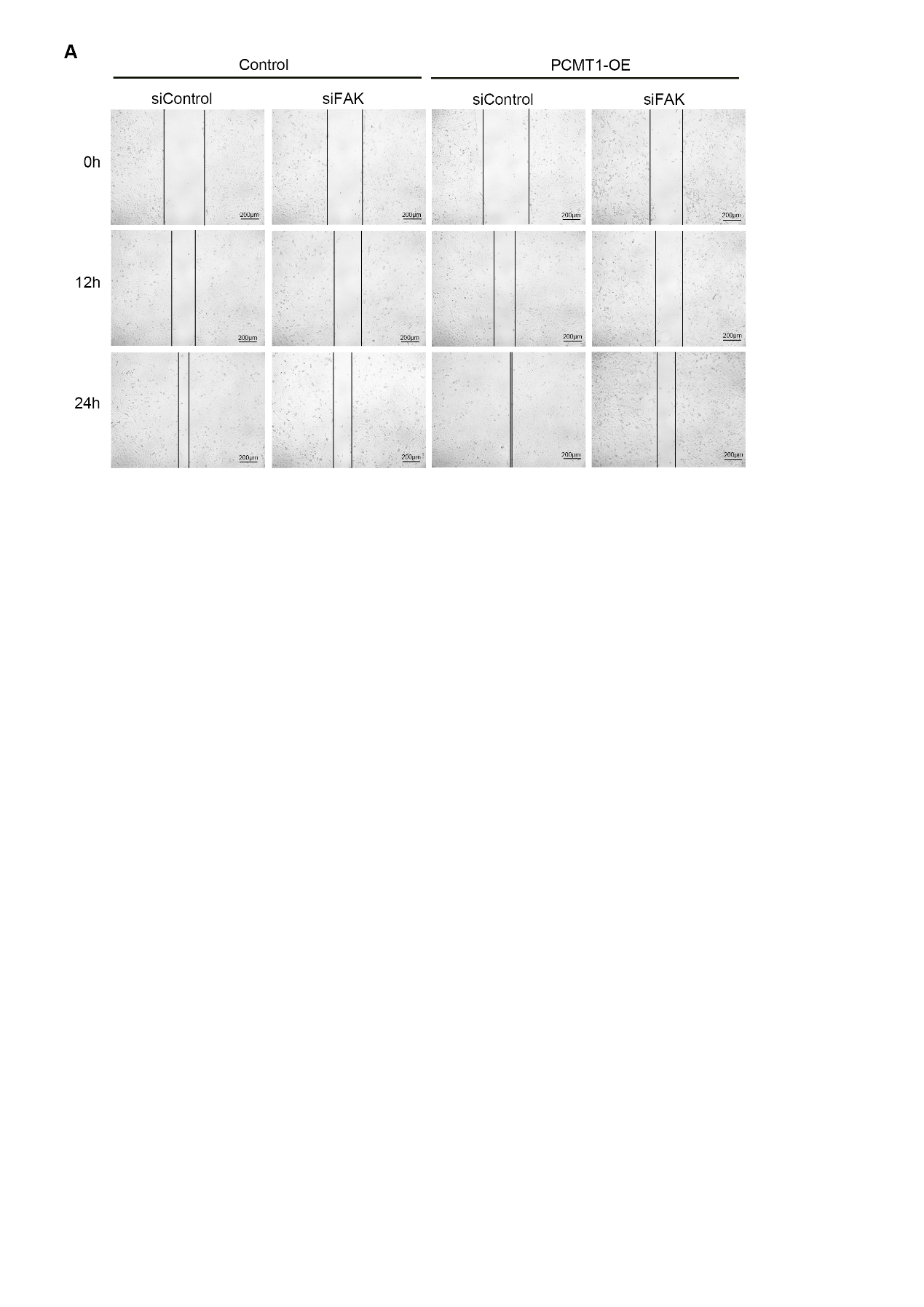


**Figure S7 *FAK* knockdown in PCMT1-OE cells inhibits cell migration.** (A) PCMT1-OE SKOV3 cells were transfected with control siRNA and FAK siRNA, and cell migration was assessed.

**Figure S8**


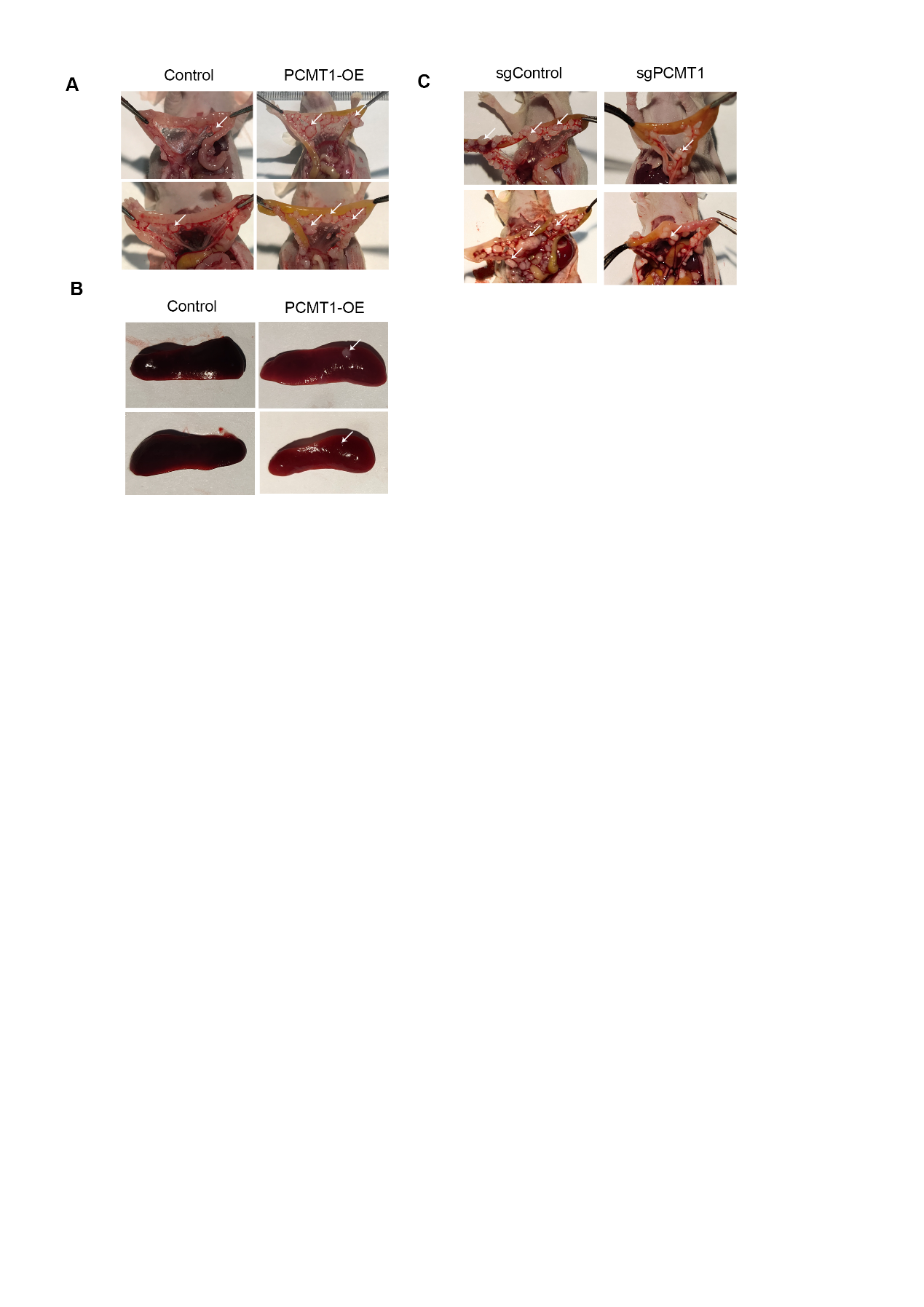


**Figure S8 PCMT1 promotes ovarian cancer cell metastasis *in vivo*.**

(A and B) Representative images of metastatic tumors in the mesenterium (A) and spleen (B) in mice injected with control or PCMT1-OE SKOV3 cells. (C) Representative images of metastatic tumors in the mesenterium in mice injected with control (sgControl) or *PCMT1*-KO (sgPCMT1) SKOV3 cells.
