# Supplementary material for: Genome-wide CRISPR/Cas9 library screen identifies PCMT1 as a critical driver of ovarian cancer metastasis
Source: J Exp Clin Cancer Res. 2022 Jan 15;41:24. doi: 10.1186/s13046-022-02242-3 (PMC8760697; doi:10.1186/s13046-022-02242-3)
Supplement: Supplementary file 2 — Additional file 2: Table S1. The siRNAs used for specific genes knockdown. Table S2. Primers used for real-time PCR amplification. Table S3. The sgRNA used for specific genes knockout.. [file 13046_2022_2242_MOESM2_ESM.docx]

**Table S1: The siRNAs used for specific genes knockdown**

| Genes | Target sequences (5’-3’) |
| --- | --- |
| siRNA-PCMT1-1  siRNA-PCMT1-2 | 5’- GCGCUAGAACUUCUAUUUGAU-3’  5’- GAUCACAUUAAAGAGCUAGUA-3 |
| siRNA-KCTD10 | 5’- GUAACAACAAAUACUCAUA-3’ |
| siRNA-ACTR10 | 5’- UGAUUAUCGAAUCGGUAUUAU-3’ |
| siRNA-LAMB3-1  siRNA-LAMB3-2 | 5’- GUGUGUGCAAGGAGCAUGU-3’  5’- GAAGCUUCAAUGGUCUCCUUA -3’ |
| siRNA-FAK  siRNA-Control | 5’- GCAUGUGGCCUGCUAUGGA-3’  5’- UUCUCCGAACGAGUCACGU-3’ |

**Table S2: Primers used for real-time PCR amplification**

| Genes | Forward primer (5’ to 3’) | Reverse primer (5’ to 3’) |
| --- | --- | --- |
| ACTR10 | 5’- GTCGTGATCGACCTGGGAGA-3’ | 5’-TGGGATTCACCAATAGATGCCT-3’ |
| FAM32A | 5’- AGGCGGCCTTCGAGAAAATG-3’ | 5’-GAATGTCGTAATGCTCCGTGA-3’ |
| KCTD10 | 5’- CGGTGCCTTTACCCGAGAG-3’ | 5’-AACTTCACGGCTGGCTTATTT-3’ |
| PCMT1 | 5’- AGGAAGGACGATCCAACACTT-3’ | 5’-GGCTTCTTCAGCATATCCCATT-3’ |
| PSMD14 | 5’- AAGTTATGGGTTTGATGCTTGGA-3’ | 5’-ATACCAACCAACAACCATCTCC-3’ |
| RBM8A | 5’- GATGGGGACGAGAGCATTCAC-3’ | 5’-CGCTGTCATAATCCTCACGCA-3’ |
| RIF1 | 5’- GCACAAAAGGTACATTTGCGG-3’ | 5’-AGCTGCTCCGTAATAGATGCT-3’ |
| TCEB1 | 5’- CATCAGGCACGATAAAAGCCA-3’ | 5’-GCTGTTAGTGTAGCGAACCTTG-3’ |
| LAMB3 | 5’- GACTTCGGTAAGACCTGGCG-3’ | 5’-CTCTCAAGTTTGTGATCTCCCC-3’ |
| ITGB1 | 5’- CCTACTTCTGCACGATGTGATG-3’ | 5’-CCTTTGCTACGGTTGGTTACATT-3’ |
| GAPDH | 5’- CTGGCCAAGGTCATCCATGAC -3’ | 5’-CTTGCCCACAGCCTTGGCAG-3’ |

**Table S3: The sgRNA used for specific genes knockout**

| Genes | Target sequences (5’-3’) |
| --- | --- |
| sgRNA-PCMT1 | 5’- GCGATGGCCTGGAAATCCGG-3’ |
